# Triphenyl phosphate promotes lipid accumulation in human mesenchymal stem cells through metabolic stress pathways

**DOI:** 10.1007/s00204-026-04445-5

**Published:** 2026-06-02

**Authors:** M. C. Gronske, W. Stutts, D. Jima, J. Palumbo, H. M. Stapleton, S. W. Kullman

**Affiliations:** 1 Comparative Biomedical Sciences, College of Veterinary Medicine, North Carolina State University, Raleigh, NC USA; 2 Molecular Education, Technology and Research Innovation Center (METRIC), North Carolina State University, Raleigh, NC USA; 3 Center for Human Health and the Environment, North Carolina State University, Raleigh, NC USA; 4 Bioinformatics Research Center, North Carolina State University, Raleigh, NC USA; 5 Toxicology Program, North Carolina State University, Raleigh, NC USA; 6 Nicholas School of the Environment, Duke University, Durham, NC USA; 7 Department of Biological Sciences, North Carolina State University, Raleigh, NC USA

**Keywords:** Triphenyl Phosphate, Mesenchymal Stem Cell, Flame Retardants, Adipogenic

## Abstract

Organophosphate esters (OPEs), used as both plasticizers and flame retardants, are increasingly recognized for their potential to disrupt cellular homeostasis and emerging evidence indicates their ability to induce metabolic stress in diverse biological systems. This study investigated the impact of Triphenyl Phosphate (TPhP) on human mesenchymal stem cell (hMSC) differentiation and cell stress. hMSCs were treated with DMSO (vehicle control), 10 µM rosiglitazone (a PPARγ agonist), or 25 µM TPhP for 14–21 days. Following treatment, cells were stained to assess nuclear integrity, lipid accumulation, and mineralization. Immunocytochemistry (ICC) for osteogenic and adipogenic markers was used to determine whether TPhP promotes adipogenesis under osteogenic conditions. TPhP exposure led to significant lipid accumulation without activation of adipogenic differentiation programs and markedly reduced mineralization. In particular, co-treatment with PPARγ inhibitors did not mitigate lipid accumulation or modulate the expression of adipogenic regulators. Gene expression analysis following TPhP exposures revealed upregulation of pathways involved in lipid biogenesis, triglyceride synthesis, phospholipid metabolism and endoplasmic reticulum (ER) stress, with minimal changes to classical adipogenic markers. These findings indicate that TPhP disrupts the metabolic balance between osteogenesis and lipid metabolism, with the potential to promote ectopic lipid deposition within the bone forming environment. This dysregulation is relevant for the development of metabolic disorders, including obesity, type 2 diabetes, and metabolic bone diseases, including osteoporosis.

**Supplementary Information:**

The online version contains supplementary material available at https://doi.org/10.1007/s00204-026-04445-5.

## Introduction

Organophosphate esters (OPEs) are a broad and chemically diverse class of additives extensively used as flame retardants and plasticizers in consumer and industrial products (Kemmlein et al. 2003; van der Veen & de Boer 2012). Since their widespread adoption in the mid-twentieth century, OPEs have been incorporated into electronics, furniture, textiles, building materials, and plastics, resulting in their pervasive presence in indoor and outdoor environments (Weil & Levchik 2008). Although initially introduced as alternatives to other flame-retardant chemistries, many OPEs are now recognized as persistent environmental contaminants with the potential to adversely affect human health (Phillips et al. 2017). Among the major subclasses of OPEs, aryl organophosphate esters (AOPEs) have become increasingly prominent, particularly following the phase out of halogenated flame retardants. AOPEs are produced in high volumes and are found across a wide range of industrial and consumer products, contributing to extensive environmental release and human exposure (Carlsson et al. 2000; Marklund et al. 2005a, 2005b; Salthammer et al. 2003; Hu et al. 2023). Human exposure to AOPEs occurs through multiple routes, including dietary intake, inhalation, dermal contact, and maternal infant transfer, with these compounds frequently detected as mixtures in indoor dust (Gbadamosi et al. 2021; Hoffman et al. 2017; Hu et al. 2023; Li et al. 2023; Zhao et al. 2016). Consistent with these exposure pathways, AOPE metabolites are commonly detected in human urine, often at higher levels in young children than in adults (Hoffman et al. 2017; Phillips et al. 2017; Gbadamosi et al. 2021; L. Wang et al. 2020). Emerging evidence further indicates that select AOPEs can disrupt metabolic and developmental pathways, underscoring concerns about their endocrine disrupting properties and potential long-term impacts on human health (Du et al. 2019).

Triphenyl Phosphate (TPhP) has been consistently reported to be one of the most prevalent organophosphate flame retardants (OPFRs) in indoor dust and in surface waters (Green et al. 2008; Stapleton et al. 2009; Zheng et al. 2015). Although TPhP is not classified as persistent or highly bioaccumulative like brominated flame retardants (BRFs), its environmental ubiquity raises concerns about chronic exposure in humans and wildlife. A growing body of evidence now links TPhP to a range of adverse health outcomes, including neurotoxicity, developmental toxicity, endocrine disruption, non-alcoholic fatty liver disease (NAFLD), obesity, and metabolic disorders (Negi et al. 2021; van der Veen & de Boer 2012; D. Wang et al. 2019; Yue et al. 2023; X. Zhong et al. 2021). In vitro, mouse BMS2 cells exposed to TPhP exhibited marked lipid accumulation, while in vivo studies demonstrate that exposure to TPhP decreases growth rates in weanling rats and alters hepatic protein profiles (Pillai et al. 2014; Hinton et al. 1987). Perinatal exposure to TPhP has also been shown to increase body and fat mass and accelerate the onset of type 2 diabetes in rats (Green et al. 2017). Furthermore, pre- and postnatal exposure to Firemaster 550, a mixture of AOPEs and BFRs, has been associated with increased anxiety, early puberty, and obesity in rodent models (Patisaul et al. 2013). Given its endocrine-disrupting properties, TPhP may play a critical role in disrupting the metabolic balance between adipogenesis and osteogenesis, contributing to long-term metabolic health risks.

Obesity has reached pandemic levels worldwide and affects individuals across all age groups. While traditionally viewed as a consequence of caloric excess and insufficient energy expenditure, growing evidence supports a more complex etiology involving environmental factors (Heindel & Blumberg 2019). In 2006, Grün and Blumberg proposed the *obesogen hypothesis*, suggesting that certain environmental endocrine-disrupting chemicals (EDCs) can disrupt normal adipocyte development and lipid metabolism, particularly during early development, thereby predisposing individuals to obesity later in life (Grün & Blumberg 2006). Obesogens are chemical compounds that disrupt normal lipid homeostasis and adipogenesis, promoting an inappropriate increase in white adipose tissue mass and fat accumulation in vivo. (Grün & Blumberg 2006; Heindel & Blumberg 2019). Among suspected obesogens, AEOPs have drawn significant attention due to their ability to disrupt lipid balance and contribute to conditions such as metabolic dysfunction, steatotic liver disease (MASLD) and metabolic syndrome (Che et al. 2023; Che et al., 2024; Jain et al. 2024).

As a metabolic organ, bone formation is influenced by a complex interplay of genetic, physiological, and environmental factors. Intrinsic determinants such as ethnicity, sex, age, and endogenous hormone levels contribute to individual variability in skeletal health and susceptibility to bone disorders (Rubio-Gutierrez et al. 2022). While osteogenesis (bone formation) and bone remodeling are governed by conserved regulatory pathways, individual differences in these inherent factors play a role in overall bone health. Simultaneously, extrinsic factors including lifestyle, nutrients, prescription drugs, and exposure to environmental contaminants can have direct effects on osteogenesis and remodeling resulting in detrimental effects on skeletal health throughout an individual’s lifespan (Rubio-Gutierrez et al. 2022). Essential for osteogenesis, mesenchymal stem cells (MSC) differentiate into a variety of mesenchymal lineages, including osteoblasts (bone cells), and adipocytes (fat cells). Both self-renewal and differentiation/fate determination of MSCs are governed by strict cross talk between epigenetic modulators and transcriptional regulators that control the regulatory networks involved in cellular differentiation and patterning of bone and adipose tissue (Lefebvre & Bhattaram 2010; Pierce et al. 2019). A growing body of evidence suggests that exposure to various chemical agents may dysregulate MSC regulatory pathways governing cellular events responsible for stem cell commitment, differentiation and/or maturation (Alexander et al. 2016; Tyl et al. 2007, Watson et al., 2019).

In this study, we investigate the role of TPhP in facilitating lipid production in human mesenchymal stem cells programmed to become osteoblasts. Previous studies suggest that TPhP functions as a PPARy agonist that promotes the differentiation of multipotent stem cells into an adipogenic lineage (Pillai et al. 2014). Here, we demonstrate that TPhP additionally induces hallmarks of stress-adaptive lipid remodeling by facilitating broad reprogramming of lipid metabolism outside of standard adipogenic programming.

## Materials and methods

### In vitro TPhP experiments

Bone marrow-derived human mesenchymal stem cells (hBMSCs) were obtained from Lonza Bioscience at passage 2 and were maintained and expanded under standard culture conditions in growth media (GM) composed of minimum essential medium, α-modification (α-MEM, Sigma-Aldrich) supplemented with 10% fetal bovine serum (FBS, Atlanta Biologicals), 2 mM L-glutamine (Genesee Scientific), 100 U/mL penicillin, and 100 µg/mL streptomycin (Genesee Scientific). hBMSCs between passages 5–7 were seeded into 12-well tissue culture treated plates (Genesee Scientific) at a density of 5 × 10^3^ cells/cm^2^ in GM. For differentiation assays, media was replaced at 48 h post-seeding with osteogenic differentiation medium (ODM) with insulin (ODMI); (Osteo-induction), composed of α-MEM supplemented with 10% FBS, 2 mM L-glutamine, 100 U/mL penicillin, 100 μg/mL streptomycin, 50 μM ascorbic acid (Sigma-Aldrich), 0.1 μM dexamethasone (Sigma-Aldrich), 10 mM β-glycerophosphate (Sigma-Aldrich), and 0.5 µg/mL human insulin (Sigma-Aldrich) containing one of the following treatments: 0.1% dimethyl sulfoxide (DMSO) (Sigma-Aldrich), 10 μM Rosiglitazone (Sigma-Aldrich), or 25 μM TPhP (Sigma-Aldrich, 99% purity) dissolved in DMSO as a vehicle. Triphenyl phosphate (TPhP) at concentration of 25 µM was selected for this study to balance biological relevance with experimental sensitivity and mechanistic resolution and remains below thresholds associated with non-specific cytotoxicity (Supplemental Fig. 1), allowing for clear interpretation of mechanistic outcomes. Negative controls included undifferentiated cells cultured in GM containing 0.1% DMSO and the Rosiglitazone treated cells served as our positive control for adipocyte differentiation. All treatments were cultured and maintained under standard culture conditions for up to 21 days and dosed media was replaced every 2 – 3 days.

**Fig. 1:**
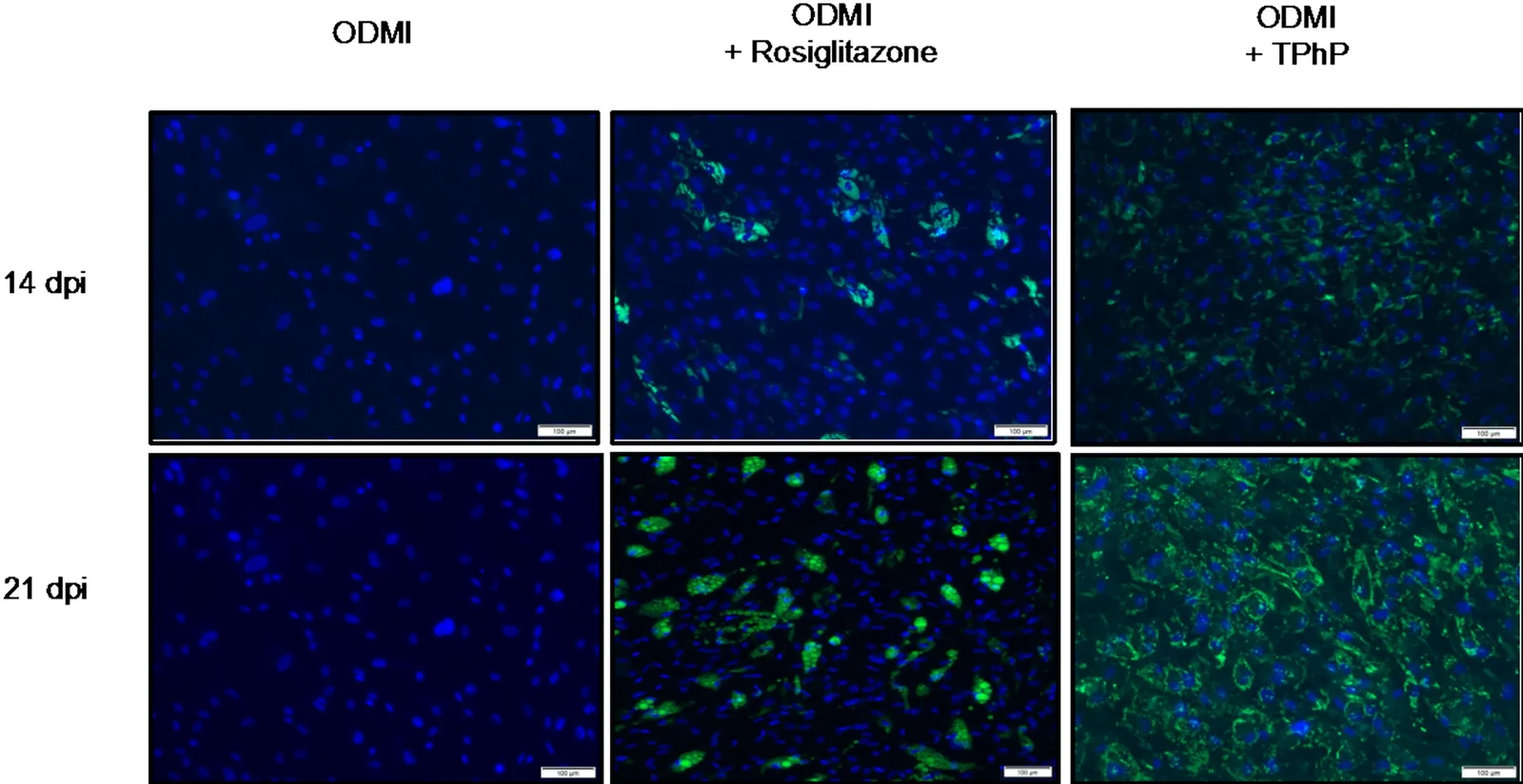
Lipid assessment of human bone marrow-derived mesenchymal stem cells (hBMSCs). hBMSCs were cultured in osteogenic differentiation media with insulin (ODMI), ODMI + Rosiglitazone (10µM), and ODMI + TPhP (25µM) for 21 days. Neutral lipid (Green) and nuclear (Blue) staining were performed on days 14 and 21

All inhibitor studies were conducted in ODMI in the presence of 10 µM Rosiglitazone or 25 µM TPhP and assessed at 14 or 21 post osteo-induction. PPARγ inhibitors GW9662 (Cayman Chemical) and T0070907 (Cayman Chemical) were used at final concentrations of 36.5 and 20 µM, respectively. Treated cells were maintained under standard culture conditions and treatment dosed ODMI was refreshed every 2–3 days. At 14 and/or 21 days, cells were fixed for 20 min at room temperature with 4% paraformaldehyde (PFA) in PBS (ThermoFisher Scientific) and stained for nuclei, neutral lipid, and phospholipid. Nuclei were stained with Invitrogen™ NucBlue Live ReadyProbes Reagent (Hoechst 33,342) (ThermoFisher Scientific). Neutral lipid was stained with 1X AdipoRed Assay Reagent (Lonza Bioscience) or 1X HCS LipidTOX Green Neutral Lipid Stain (ThermoFisher Scientific). Phospholipid was observed with 1X HCS LipidTOX™ Red Phospholipidosis Detection Reagent (ThermoFisher Scientific). Cells were imaged using a Zeiss LSM 880 confocal microscope (Carl Zeiss Microscopy GmbH, Jena, Germany) with ZEN 2012 version 2.3 SP1 Image Acquisition Software (Carl Zeiss) at 10X magnification.

### Measurement of extracellular matrix

Cell monolayers were stained with Alizarin Red S (ARS) at 14 days post osteo-induction to label mineralized extracellular matrix. Briefly, cells were washed twice in PBS, fixed in 10% neutral buffered formalin for 20 min, washed twice with dd H_2_0, and stained for 15 min with 40 mM Alizarin Red S (Sigma-Aldrich). Stained monolayers were washed five times in ddH_2_0 and representative wells were imaged. Mineralization was quantified using the Osteogenesis Quantitation Kit (Millepore). After imaging, 800 µL of 10% acetic acid was added to each well and incubated for 30 min before the contents of each well was harvested by scraping. Samples were heated to 85 °C for 10 min then cooled on ice. Samples were centrifuged at 20,000 × g for 15 min, and the supernatants were transferred to an opaque-walled clear, flat-bottom 96-well plate. Optical density was measured in a FLUOstar spectrophotometer at 405 nm and Alizarin Red concentration was derived from a standard curve using linear regression analysis.

### Immunocytochemistry

hBMSCs were induced in ODMI and were simultaneously exposed to 0.1% DMSO, 10 µM Rosiglitazone, or 25 µM of TPhP. To visualize cells differentiated to adipocytes in each exposure group, cells were fixed after 21 days of growth with 4% PFA or methanol for 20 min at room temperature then washed and stored in 1X PBS at 4 ˚C. Cells were permeabilized in 0.25% Triton X-100 in 1X PBS for 15 min at room temperature. Cells were then blocked in 2% BSA and 0.1% Tween-20 in 1X PBS for 3 h at room temperature. The conjugated primary antibody for *perilipin-1* (*PLN*) (Perilipin Antibody (Alexa Fluor® 405), Novus Biologicals) was diluted to 5 μg/mL in dilution buffer composed of 1% BSA and 0.3% Tween-20 in 1X PBS before addition to the cells for overnight incubation at 4 ˚C. *Perilipin-*1 is a lipid droplet coating protein involved in the lipid metabolism of adipocytes. Cells were washed the following day with 1X PBS then counterstained with NucBlue and Invitrogen™ HCS LipidTOX Deep Red Neutral Lipid Stain (ThermoFisher Scientific) for 30 min before imaging under the confocal microscope at 10X magnification.

For ICC staining of methanol fixed osteoblasts, cells were permeabilized for 10 min in 0.1% Triton X-100 in 1X PBS at room temperature. Cells were then blocked in 2% BSA in 1X PBS for 1 h at room temperature. The unconjugated primary antibody for *distal-less homeobox 5 (DLX5* polyclonal antibody rabbit anti-human) (ThermoFisher Scientific) was diluted to 2 μg/mL in antibody dilution buffer (1% BSA in 1X PBS) before addition to the cells for overnight incubation at 4 ˚C. *Distal-less homeobox 5* is a transcription factor involved in bone development. Cells were washed the following day with 1X PBS then incubated for 45 min at room temperature in the dark in a secondary antibody (goat anti-rabbit 488 green) (ThermoFisher Scientific) diluted to 2 μg/mL in antibody dilution buffer. Cells were then washed with 1X PBS and counterstained with NucBlue and 1X HCS LipidTOX Deep Red Neutral Lipid Stain (ThermoFisher Scientific) stain 30 min before imaging on the confocal microscope at 10X magnification.

### RNA isolation and qPCR

At 14 and 21 days, cells were washed twice with ice cold 1X PBS to remove any remaining media. 700 µL of TRI Reagent (Invitrogen) was then added to each well. Cells were scraped from the wells and the mixture from each well was transferred to separate microcentrifuge tubes. Total RNA was isolated according to the protocol described by the TRI Reagent manufacturer. RNA was then quantified using the Qubit® RNA HS Assay Kit (Invitrogen) containing Qubit® RNA HS Reagent and Buffer with the Qubit® 3.0 Fluorometer.

cDNA was synthesized from 1µg of total RNA using High-Capacity cDNA Reverse Transcription Kit (Applied Biosystems, Foster City, California, USA) containing random primers, MultiScribe Reverse Transcriptase, RNase inhibitor, deoxynucleotide triphosphate mix, and 10X reverse transcription buffer in a 20 μL reaction. Human-specific real time PCR primers were designed in Primer3 and procured from IDT (Integrated DNA Technologies) (Supplemental Tables. 1–4). To quantify relative gene expression, cDNA from DMSO- and TPhP-treated samples (n = 3–4) were PCR amplified in quadruplicate on clear, 96-well PCR plates (Olympus Plastics, Genesee, San Diego, California, USA) using a QuantStudio 3. Each 20 μL qPCR reaction was comprised of 10 μL of SYBR Green real time PCR master mix (Life Technologies), 7.6 μL of UltraPure Distilled H_2_O (Life Technologies), 0.4 μL of 10 μM forward primer, 0.4 μL of 10 μM reverse primer, and 1.6 μL of cDNA. The conditions for each PCR reaction were as follows: (1) 50 °C for 2 min, (2) 95 °C for 10 min, (3) 95 °C for 15 s followed by 60 °C for 60 s, repeated 40 × , and (4) 95 °C for 15 s, 60 °C for 60 s, 95 °C for 15 s, and 60 °C for 15 s to derive dissociation or melt curves and ensure specificity for each primer set used. Threshold cycle (C_t_) values for each reaction were determined by the QuantStudio™ Design & Analysis Software (Applied Biosystems, v1.5.2) and relative gene expression was determined according to the ΔΔC_t_ method (Livak & Schmittgen 2001) by normalizing each gene to *RPL13α*.

**Table 1:** Bulk RNA Seq D21 adipocyte related gene expression

Gene	Rosiglitazone fold change	Rosiglitazone padj	TPhP fold change	TPhP padj	Ensembl ID	Description
PLIN4	194.33	0	5.90	4.18292E-62	ENSG00000167676	Perilipin 4
FABP4	165.10	2.85043E-85	3.98	2.06787E-20	ENSG00000170323	Fatty acid binding protein 4
ADIPOQ	74.65	1.7263E-100	2.76	3.34457E-15	ENSG00000181092	Adiponectin, C1Q and collagen domain containing
PLIN1	19.45	7.73367E-54	2.48	5.59036E-09	ENSG00000166819	Perilipin 1
LIPE	14.11	5.5038E-85	0.92	0.685374736	ENSG00000079435	Lipase E, hormone sensitive type
CD36	12.05	4.06029E-44	1.26	0.000229435	ENSG00000135218	CD36 molecule
LPL	11.33	5.76167E-36	1.40	0.002992783	ENSG00000175445	Lipoprotein lipase
DGAT2	8.63	2.41166E-51	0.20	0.248216391	ENSG00000062282	Diacylglycerol O-acyltransferase 2
CIDEC	5.34	2.2545E-39	1.26	0.062180334	ENSG00000187288	Cell death inducing DFFA like effector c
FABP3	4.98	1.17159E-32	1.26	0.178447178	ENSG00000121769	Fatty acid binding protein 3
FABP5	4.90	3.94492E-77	1.27	0.032216315	ENSG00000164687	Fatty acid binding protein 5
PLIN2	4.47	2.89262E-25	1.31	0.138838636	ENSG00000147872	Perilipin 2
CEBPA	4.28	2.55404E-23	1.10	0.649180626	ENSG00000245848	CCAAT enhancer binding protein alpha

### Lipidomics

 Human MSCs were seeded at a density of 25,000 cells per well and cultured in growth media for 48 h. Differentiation was then initiated using osteo-induction medium (ODMI) supplemented with either 0.1% DMSO (vehicle control), 10 µM ROSI, or 25 µM TPhP. Following a 21-day differentiation period, media was aspirated and the adherent cells were washed in ice cold PBS, frozen on ice, and harvested by scraping cells in cold 80:20 (v/v) methanol:water. Samples were stored at −80 °C until further processing. For each of the four biological replicates per treatment, cells from nine individual wells were pooled and processed for lipid metabolites using a modified Folch protocol (Folch et al., 1957). Briefly, samples were Spike with 10uL Avanti Splash-Lipidomic standards (Avanti, Alabaster, Alabama) and extracted with cold chloroform, incubated on ice for 30 min and vortexed in the presences of cold water. The lower organic phase was transferred to a new test tube and the aqueous layers were reextracted with 1:2 methanol:chloroform. Samples were dried under N_2_ and the organic phase was reconstituted in isopropyl alcohol. Lipids were separated on an Acquity Premier CSH C18 column (1.7µm, 2.1 × 100 mm) and untargeted lipidomics data were acquired in positive and negative ionization modes using a Thermo Scientific™ Orbitrap ID-X Tribrid MS coupled to a Vanquish UHPLC. Compound Discoverer (CD) 3.3 SP2 and LipidSearch (LS) 5.1 were used for data analysis including compound annotations with mzCloud, mzVault and LS alignment data exported to mass lists for final compound discovery.

### Bulk RNASeq-method and analysis

Total RNA samples from four replicates of each 0.1% DMSO (control), 10 µM ROSI or 25 µM TPHP treated hMSCs were submitted to the North Carolina State University Genomic Sciences Laboratory (Raleigh, NC, USA) for Illumina RNA library construction and sequencing. Library construction, RNA integrity, purity, and concentration were assessed using an Agilent 2100 Bioanalyzer with an RNA 6000 Nano Chip (Agilent Technologies, USA). Purification of messenger RNA (mRNA) was performed using the oligo-dT beads provided in the NEBNext Poly(A) mRNA Magnetic Isolation Module (New England Biolabs, USA). Complementary DNA (cDNA) libraries for Illumina sequencing were constructed using the NEBNext Ultra Directional RNA Library Prep Kit (NEB) and NEBNext Mulitplex Oligos for Illumina (NEB) using the manufacturer-specified protocol. Briefly, the mRNA was chemically fragmented and primed with random oligos for first strand cDNA synthesis. Second strand cDNA synthesis was then performed with dUTPs to preserve strand orientation information. The double-stranded cDNA was then purified, end-repaired and “a-tailed” for adaptor ligation. Following ligation, the samples were selected for a final library size (adapters included) of 400–550 bp using sequential AMPure XP bead isolation (Beckman Coulter, USA). Library enrichment was performed and specific indexes for each sample were added during the protocol-specified PCR amplification. The amplified library fragments were purified and checked for quality and final concentration using an Agilent 2200 Tapestation with a High Sensitivity DNA chip (Agilent Technologies, USA) and a Qubit fluorometer (ThermoFisher, USA). The final quantified libraries were pooled in equimolar amounts for clustering and sequencing on an Illumina HiSeq 2500 DNA sequencer utilizing a 125 bp single-end sequencing reagent kit (Illumina, USA). The software package Real-Time Analysis (RTA) was used to generate raw bcl, or base call files, which were then de-multiplexed by sample into fastq files for data submission. All RNA-Seq data have been deposited in GEO.

### RNA seq data analysis

Bioinformatics processing and data interpretation were carried out in consultation with the Bioinformatics Core at the NCSU Center for Human Health and the Environment. The quality of sequenced data was assessed using FastQC and poor-quality bases were trimmed from the 5’-end. The remaining good quality reads were aligned to the Human reference genome (hg38)using STAR aligner (Dobin et al., 2013). For each replicate, per-gene counts of uniquely mapped reads were calculated using *htseq-count* script from the HTSeq python package. We imported the count matrix to the R statistical computing environment (V 4.5.1) for further analysis. Gene count data was normalized for sequencing depth and distortion, and dispersion was estimated using DESeq2 Bioconductor package (V 1.40.2) in the R statistical computing environment (Love et al. 2014). We fitted a linear model using the treatment levels, and differentially expressed genes were identified after applying multiple testing corrections using the Benjamini-Hjockberg procedure (padj < 0.05) (Benjamini Y, Hochberg, 1995). The differential expression analysis consisted of two sets of contrasts: DMSO vs. Rosiglitazone and DMSO vs. TPhP following 21 days of cell differentiation in ODMI. Gene set enrichment analysis (GSEA (version 4.0.3)) software (Subramanian et al. 2005)⁠ was used to show statistically significant differences between DMSO and TPhP treatments (Subramanian et al. 2005.) Briefly, we pre-ranked all genes that were measured using RNA-Seq using fold change * −*log10(p value)* and sorted in descending order. Enrichment analyses were conducted against curated C2 all version 7.1 symbols. In the GSEA application we modified the following parameters (Enrichment statistics: classic, exclude large sets: 5000, exclude smaller sets: 15). We then assessed the top 20 differentially enriched gene sets and chose to present the most relevant gene set based on our described phenotypes.

### Statistical analysis

Results for the assays described above are presented as the mean ± SD (n = 3–4 technical replicates). Pairwise comparisons between ODMI-DMSO, ODMI-Rosiglitazone or ODMI-TPhP were conducted using an unpaired, one-tailed Student’s t-test with Welch’s correction. Comparisons between two or more conditions were analyzed using a One-Way Analysis of Variance (ANOVA) with a Tukey’s posthoc test to determine significant differences between multiple conditions. Significance was determined at* P*-values of either, < 0.001, < 0.05 or < 0.1 with *P*-values as indicated in each figure legend.

## Results

### TPhP exposure elicits lipid production and attenuates mineralization under osteogenic conditions

hBMSCs were induced in osteogenic differentiation medium (ODMI) and were simultaneously exposed to either 0.01% DMSO, 10 µM Rosiglitazone, or 25 µM of TPhP. Cells were incubated under normal cell culture conditions and grown out to 14 or 21 days (Fig. 1). After 14 days of exposure, cells in the DMSO control group exhibited little to no lipid droplets, as indicated by a lack of neutral lipid staining. Rosiglitazone treatment resulted in cells that contained multiple large, unilocular, refractile lipid droplets with in the cell cytoplasm, similar to that of cells that have differentiated to adipocytes (Trayhurn 2009). TPhP exposed cells produced atypical, punctate, irregularly shaped and diffuse lipid droplets that dispersed throughout the cell, with lipid seemingly produced by most cells. There was a marked absence of large coalesced lipid droplets compared to those observed with Rosiglitazone treatment and lipid appeared to be distributed within the ER and nuclear membrane of the cell. After 21 days of exposure, the Rosiglitazone group further exhibited large cells packed with abundant lipid droplets consistent with an adipocyte-like phenotype. The TPhP treated cells exhibited more abundant and pronounced diffuse lipid than observed at 14 days, appearing as small, un-nucleated bright nascent lipid droplets uncharacteristic to adipocytes (Pol et al. 2014). No overt cytotoxicity was observed following a 21-day exposure with 0.01% DMSO, 10 µM Rosiglitazone, or 25 µM of TPhP. All treatments retained 90–98% cell viability (Supplemental Fig. 1).

**Fig. 2:**
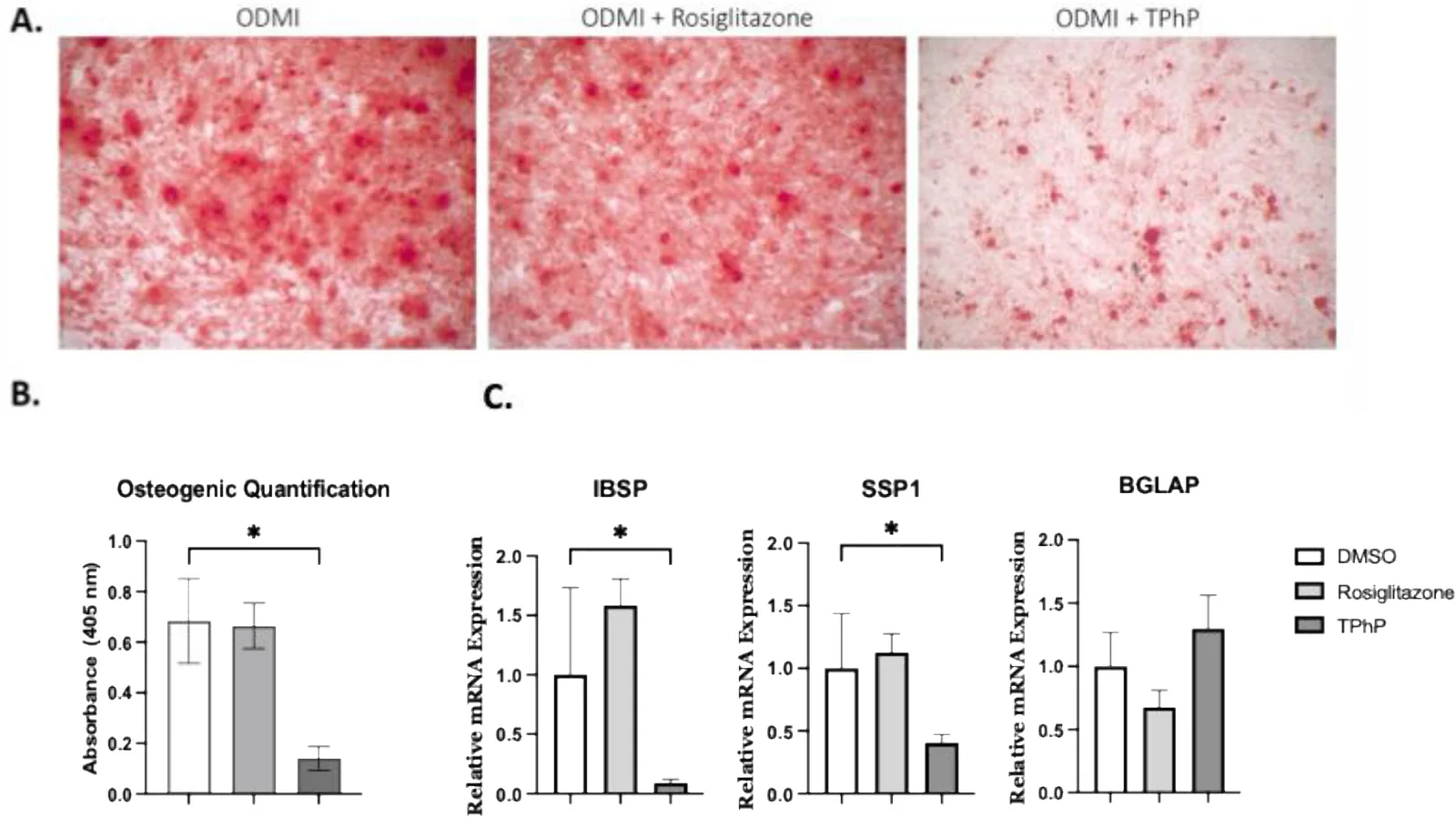
Qualitative and quantitative assessment of mineralization. Apical assessment of hBMSCs treated with ODMI, ODMI + 10 µM Rosiglitazone, and ODMI + 25 µM TPhP.** A** Alizarin Red S staining was conducted at 14 dpi to evaluate mineralization potential in vitro. **B** Mineralization was then quantified and normalized to total protein. Data represent mean ± SEM (*n* = 3–4 technical replicates). Significantly increased or decreased mineralization based on ODMI vs ODMI + Rosiglitazone and ODMI vs ODMI + TPhP is denoted by * (Student’s t-test,* p*-value < 0.05).** C** Expression of extracellular matrix genes. Asterisks denote statistical significance analyzed by Student’s T test (*P* < 0.05), based on ODMI vs ODMI + Rosiglitazone and ODMI vs ODMI + TPhP

To determine any changes to mineralization, cells were fixed after 14 days of growth and stained with Alizarin Red S (Fig. 2A). hBMSCs treated with DMSO exhibited robust mineralization throughout all well replicates indicative of rapid differentiation of MSCs to osteoblasts-like cells with the ability to secrete complex mineralized extracellular matrix. hBMSCs cells exposed to Rosiglitazone exhibited a similar ARS staining phenotype with deposition of significant extracellular matrix. Comparatively, hBMSCs exposed to TPhP exhibited a noticeable attenuation in ARS staining throughout replicate wells. Quantification of ARS staining per treatment group indicates a significant attenuation in ARS staining within the TPhP treatment groups compared to both DMSO and ROSI treatments (Fig. 2B). Consistent with a reduction in ECM, qPCR analysis demonstrated a significant attenuation in expression of *IBSP* (0.091-fold) and *SSP1* (0.42-fold) following TPhP exposure (Fig. 2C, Supplemental Table 5 for expression values and SEM for all qPCR experiments). Together, these results demonstrate that TPhP reduced the amount of extracellular mineral produced by hBMSCs that undergo osteogenesis.

### TPhP exposure elicits lipid production rather than adipocyte differentiation under osteogenic conditions

**Fig. 3:**
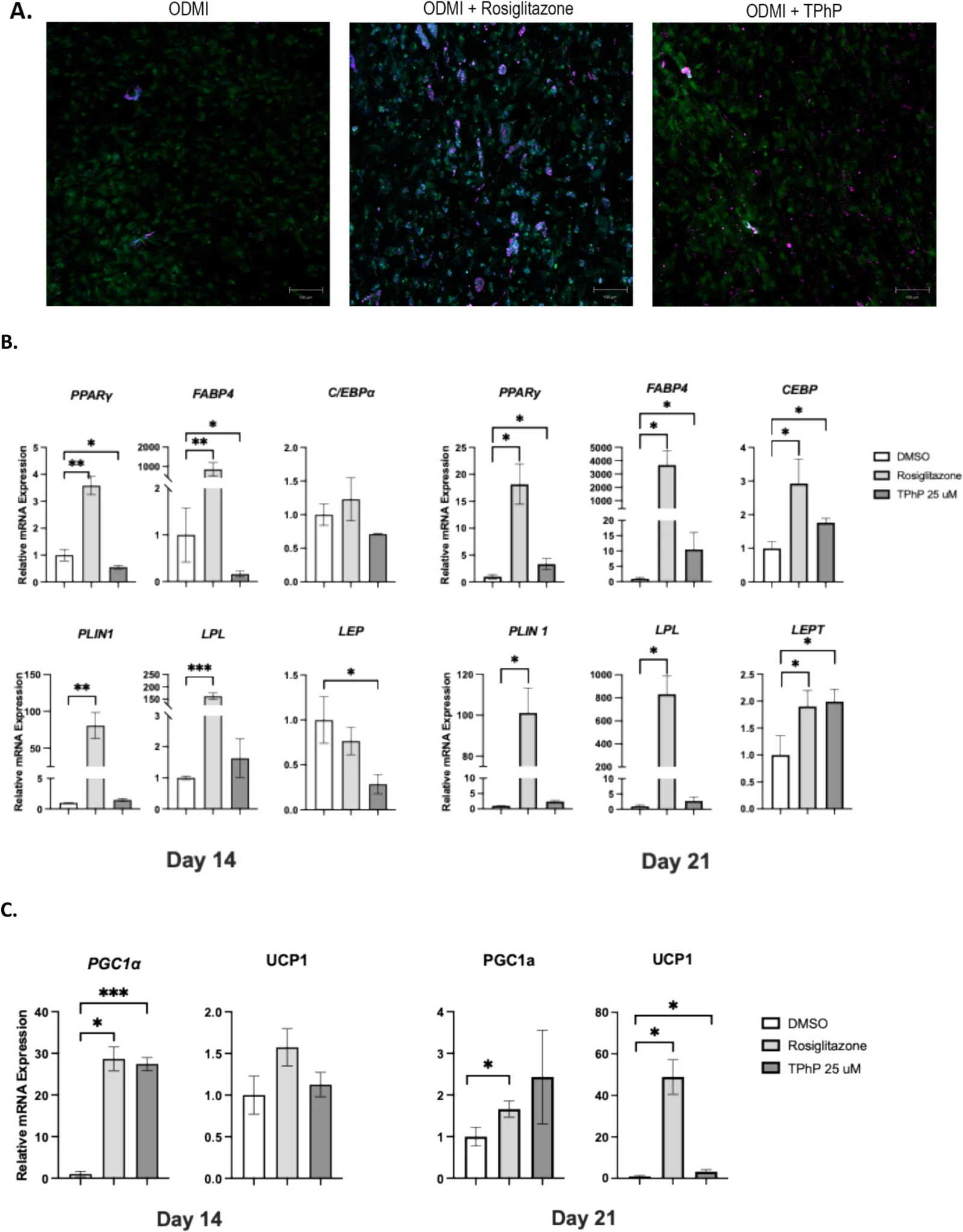
Adipogenic potential of TPhP compared to Rosiglitazone. hBMSCs were cultured in ODMI + DMSO, ODMI + Rosiglitazone (10µM), and ODMI + TPhP (25µM) for 14–21 days. **A** Immunocytochemistry (ICC) staining for an adipogenic maker, perilipin1 (*Plin1*) (Blue), was performed at day 21 and counterstained with lipid (Magenta) and nuclear stains (Green). *Plin1* coats lipid storage droplets in adipocytes. (**B**,** C**) RNA was collected on days 14 and 21 and RT-qPCR was performed to measure expression changes in genes related to adipogenesis (**B**) and the induction of mitochondrial biogenesis related to brown adipose tissue (BAT) (**C**). *** denotes statistical significance analyzed by Student’s T test  (p<0.001); **(*P* < 0.05) and *(*P* < 0.10) based on ODMI vs ODMI + Rosiglitazone and ODMI vs ODMI + TPhP

hBMSCs were induced in osteogenic differentiation (ODMI) and were simultaneously exposed to 0.1% DMSO, 10 µM Rosiglitazone, or 25 µM of TPhP. To visualize hMSCs that had undergone adipogenic differentiation across exposure groups, cells were fixed after 21 days in culture and evaluated by immunocytochemistry (ICC) using perilipin-1 (PLN1) as an adipocyte marker and counter stained with NucBlue and a deep red neutral lipid stain. (Fig. 3A). *Perilipin-*1 is a lipid droplet coating protein involved in the lipid metabolism of adipocytes (Greenberg et al. 1991). Confocal imaging of DMSO treated hBMSCs indicated minimal staining of *PLN1*, mainly highlighting the presence of a few background adipocytes, which were also visualized with the presence of larger lipid droplets. The Rosiglitazone treated hBMSCs exhibited obvious and abundant *PLN1* staining surrounding the large lipid droplets concurrent with those formed in adipocyte cell differentiation. Conversely, the TPhP treated hBMSCs exhibited the punctate lipid vesicles in the absence of *PLN1* labeling indicating these cells had not differentiated to adipocytes but rather produced abundant, diffuse neutral lipid that did not nucleate into large lipid droplets. A small degree of *PLN1* staining was observed in a few larger, adipocyte-like lipid droplets suggesting the formation of some adipocytes by 21 days post differentiation however the frequency was consistent with the DMSO background.

RT-qPCR was conducted to measure changes in expression of critical genes associated with adipocyte differentiation and mitochondrial biogenesis. hBMSCs were treated with either 0.1% DMSO, Rosiglitazone 10 µM or TPhP 25 µM and differentiated in ODMI for 14-and 21-days (Fig. 3B). At 14 days post osteoinduction, the Rosiglitazone treatment group exhibited significant upregulation of *PPARγ (3.6-fold), FABP4 (845.2-fold), PLN1 (18.6-fold), LPL (162.6-fold)* and *PGC1α (28.7-fold),* compared to the DMSO control group. TPhP treated cells exhibited elevated expression of *PGC1α* (*27.4-fold*)*,* with little induction of classic adipocyte markers compared to the DMSO control. Gene expression at 21 days post osteo-induction exhibited similar trends with Rosiglitazone inducing a robust adipogenic expression profile including *PPARγ (18.1-fold), FABP4 (3674.9-fold), PLN1 (101.2-fold), LPL (832.2-fold)* as compared to controls whereas TPhP exposures resulted in a modest induction of the adipogenic compliment comprised of *PPARγ (3.3-fold), FABP4 (10.6-fold), PLN1 (2.4-fold), LPL (2.8-fold).* Expression of *PGC1α* was significantly attenuated for both treatments compared to day 14 with a 1.7-fold and 2.4-fold induction for Rosiglitazone and TPhP respectively. Additionally, Rosiglitazone treatment resulted in a 48.8-fold induction of UCP1 at d21 while expression of UCP1 with TPhP treatment was 3.2-fold.

Bulk RNA Seq at 21 days post osteoinduction demonstrated a consistent trend in gene expression, with Rosiglitazone treated hBMSCs exhibiting a significant increase in the expression of *FABP4 (165-fold)*, *ADIPOQ (74.6-fold)*, *PLIN1 (19.5-fold)*, *LPL (11.3-fold)*, in addition to several other adipogenic related genes. By comparison, THP treatment group exhibited modest gene induction of *FABP4 (5.9-fold)*, *ADIPOQ (2.8-fold)*, *PLIN1 (2.5-fold)*, *LPL (1.4-fold)*, and other adipogenic related genes (Table 1). Gene set enrichment analysis of Rosiglitazone treatment indicted positive enrichment score for Fatty Acid Metabolism, Oxidative Phosphorylation, E2f Targets, Adipogenesis and Myc targets, and negative enrichment score for Epithelial Mesenchymal Transition, Apical Junction, and Myogenesis. Gene set enrichment analysis of TPhP treatment resulted in positive enrichment score for Protein Secretion, UV Response Down, Kras Signaling Up, Androgen Response, Hypoxia, and Complement, and negative enrichment sore for DNA Repair, UV Response Up, Interferon Alpha Response, Oxidative Phosphorylation (Supplemental Figs. 2 and 3).

**Fig. 4:**
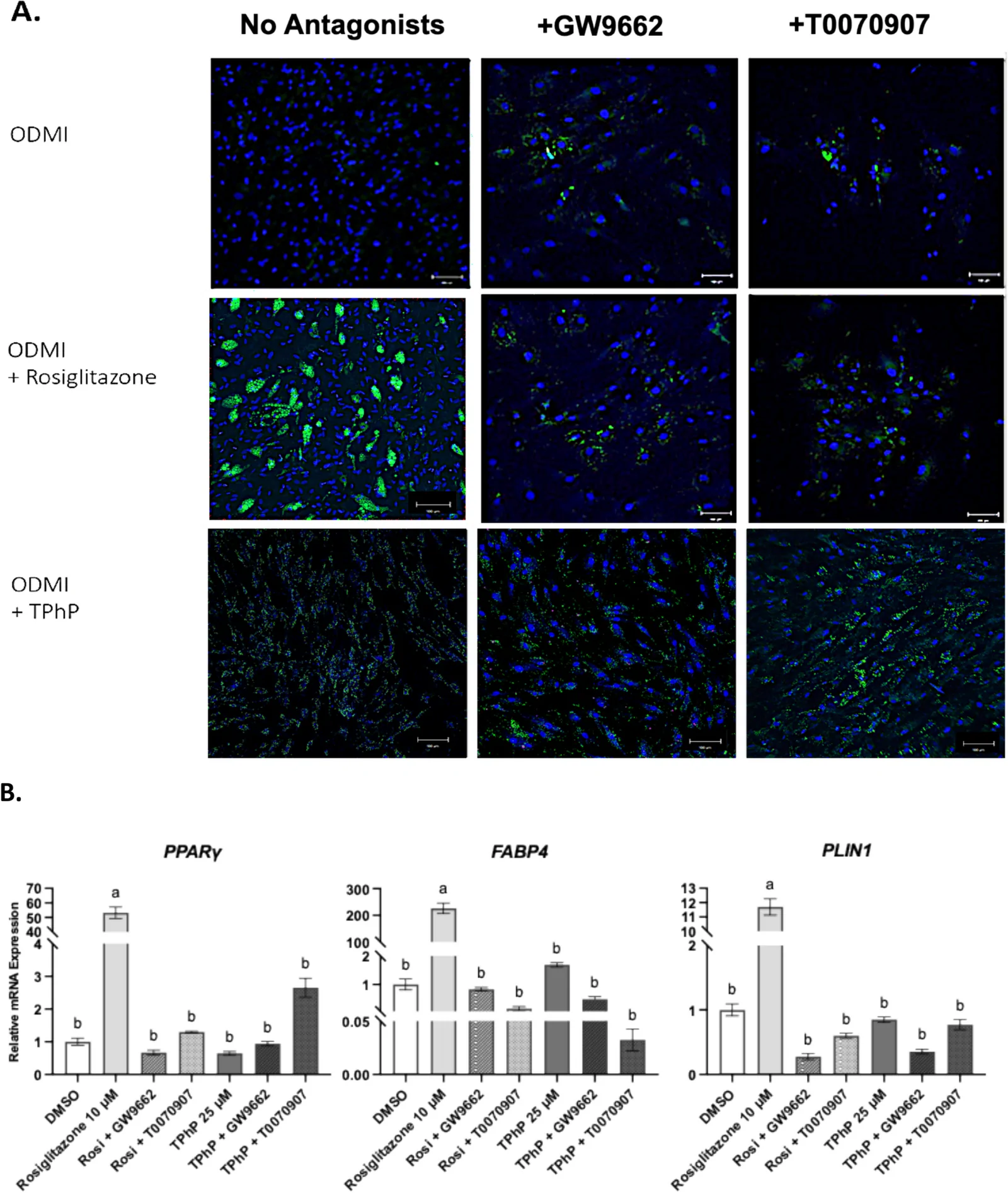
Lipid assessment with co-exposure to two different PPARγ inhibitors. hBMSCs were cultured in osteogenic differentiation media with (ODMI) + DMSO, ODMI + Rosiglitazone (10µM), ODMI + Rosi (10µM) + T0070907 (20µM), ODMI + Rosi (10µM) + GW9662 (36.5µM), ODMI + TPhP (25µM), ODMI + TPhP (25µM) + T0070907 (20µM), and ODMI + TPhP (25µm) + GW9662 (36.5µM) for 14 days. **A** Neutral lipid (Green) and nuclear staining (Blue) were performed on day 14. **B** RNA was collected at 14 dpi and RT-qPCR was performed to measure expression changes in *PPARγ, FABP4* and *PLIN1* as adipogenic gene markers. Letters denote statistical significance between groups analyzed by One-way ANOVA (*P* < 0.05)

To determine whether TPhP lipid production occurs via the activation of *PPARγ*, hBMSCs were induced in ODMI and simultaneously exposed to either 0.1% DMSO, 10 µM Rosiglitazone, or 25 µM of TPhP in the presence of one of two *PPARγ* inhibitors: T0070907 (20 µM) or GW9662 (36.5 µM) (Fig. 4). At fourteen days post osteoinduction (14dpi, to minimize putative cytotoxicity with prolonged exposure to *PPARγ* inhibitors), BMSCs were fixed in 4% PFA and stained with NucBlue and AdipoRed lipid stain to provide a qualitative, visual assessment of lipid production under each treatment condition. hBMSCs exposed to DMSO alone or DMSO with either *PPARγ* inhibitor (T0070907, GW9662) exhibited low-level lipid droplet production in a few cells, indicative of background lipid production. hBMSCs exposed to Rosiglitazone alone exhibited robust formation of cells containing unilocular lipid droplets, confirming this treatment facilitates adipocyte differentiation under osteogenic conditions. Dual treatment with Rosiglitazone and GW9662 or Rosiglitazone and T0070907, greatly attenuated adipocyte differentiation and subsequent lipid droplet formation and while few cells still exhibited some lipid droplet production, droplets were significantly smaller and did not exhibit the typical unilocular phenotype. Comparatively, hBMSCs exposed to TPhP alone exhibited diffuse punctate, lipid droplets within most cells throughout the well. hBMSCs exposed to TPhP and either *PPARg* inhibitor, GW9662 or T0070907, while slightly attenuated retained diffuse, punctate lipid droplet production in most cells throughout the well. The lipid phenotype in these treatments was consistent with that of TPhP in the absence of inhibitors suggesting lipid formation was likely not mediated through classical *PPARγ* signaling pathway. RT-qPCR was subsequently conducted to measure changes in expression of select genes that regulate adipocyte differentiation. At 14 days post osteoinduction, hBMSCs co-exposed to Rosiglitazone in the presence of *PPARγ* inhibitors exhibited significant attenuation in adipogenic-associated genes including *PPARγ*, *FABP4* and *PLIN1* (Fig. 4B). Contestant with the presence of punctate lipid droplet morphology, no statistically significant gene induction was observed with *PPARγ*, *FABP4* and *PLIN1* when hBMSC were treated with TPhP or TPhP in the presence of *PPARγ* inhibitors.

**Fig. 5:**
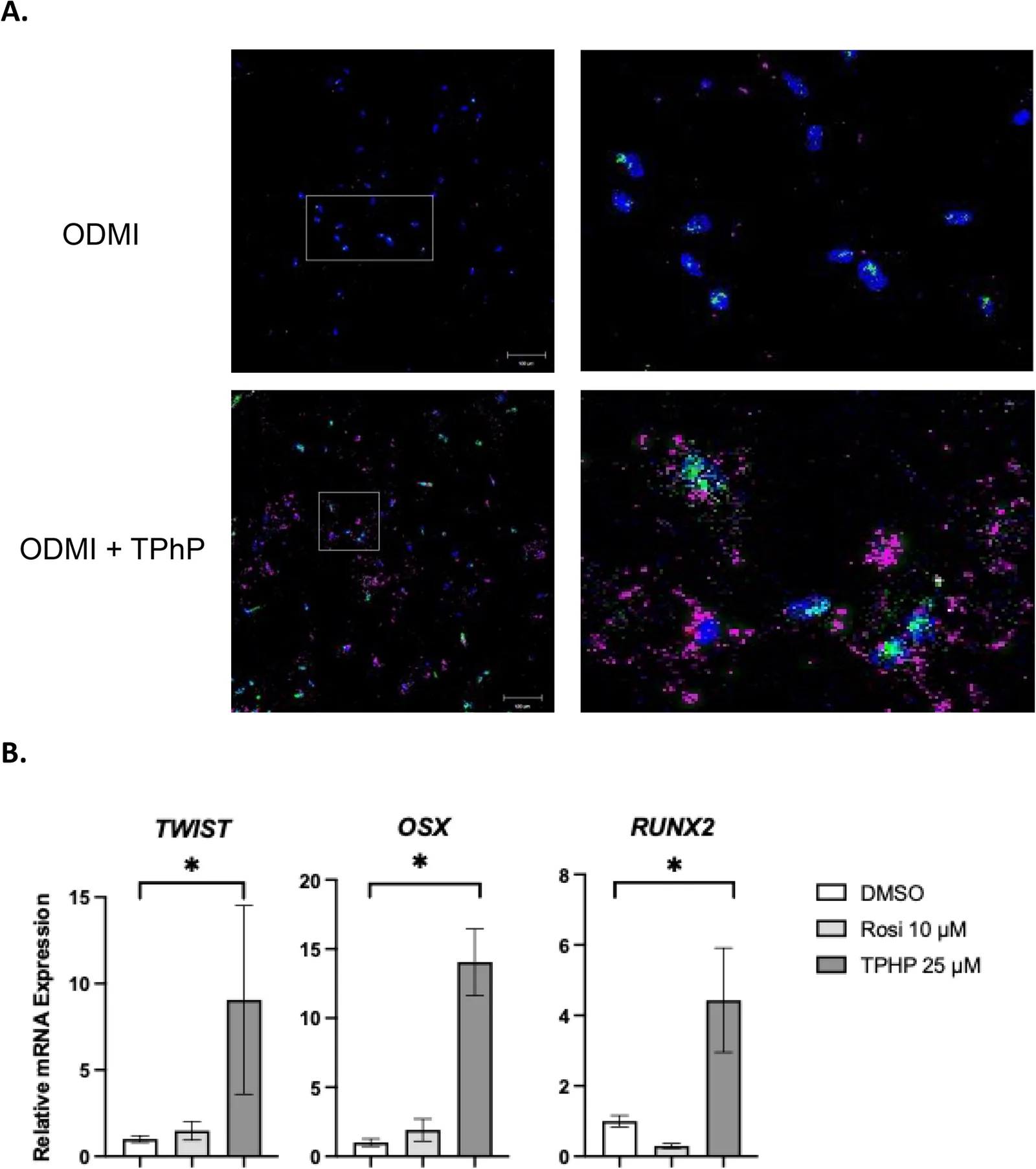
Osteogenic differentiation under osteogenic conditions. hBMSCs were cultured in ODMI + DMSO, and ODMI + TPhP (25 µM) for 14 days. **A** Immunocytochemistry (ICC) staining for an osteogenic maker, distal-less homeobox 5 (*DLX5)* (Green), was performed at day 14 and counterstained with lipid (Magenta) and nuclear stains (Blue). *DLX5* plays a role in bone development.** B** RNA was collected at 14 dpi and RT-qPCR was performed to measure expression changes in osteogenic transcription factors. Asterisks (*) denotes statistical significance analyzed by Student’s T test (*P* < 0.05) based on ODMI vs ODMI + Rosiglitazone and ODMI vs ODMI + TPhP

To assess if the cells producing lipid following TPhP treatment were differentiated osteoblasts, hBMSCs were induced in ODMI and cultured for 14 days. Cells were subsequently fixed in methanol and probed via immunocytochemistry for qualitative visualization of *distal-less homeobox 5 (DLX5)* as an osteoblast marker (Heo et al. 2017). Cells were then counterstained with NucBlue and a deep red neutral lipid stain and imaged using confocal microscopy (Fig. 5A). In hBMCs treated with DMSO, *DLX5* was abundantly visualized in the nucleus of cells throughout the field of view. Interestingly, despite the decrease in mineralized ECM production after TPhP treatment, *DLX5* was present in the nuclei of many of these cells, indicating a significant differentiation of hBMSCs towards osteoblast-like cells. Deep red neutral lipid stain was additionally present in several osteoblasts positive for *DLX5* suggesting that select osteoblasts were producing diffuse, punctate lipid in the absence of an adipocyte phenotype. Furthermore, RT-qPCR was conducted to measure changes in the expression of genes associated with osteogenic differentiation. At 14 days post osteoinduction, Rosiglitazone treated hBMSCs exhibited a significant decrease in the expression of *RUNX2* (0.3-fold) and no change in the other osteogenic genes measured compared to the DMSO control group (Fig. 5B). The TPhP treated group exhibited significant induction of *TWIST* (9.1-fold), *OSX* (14-fold) and *RunX2* (4.4-fold) compared to the DMSO control group (Fig. 5B).

### TPhP activates alternative lipid metabolic pathways beyond adipogenesis

**Fig. 6:**
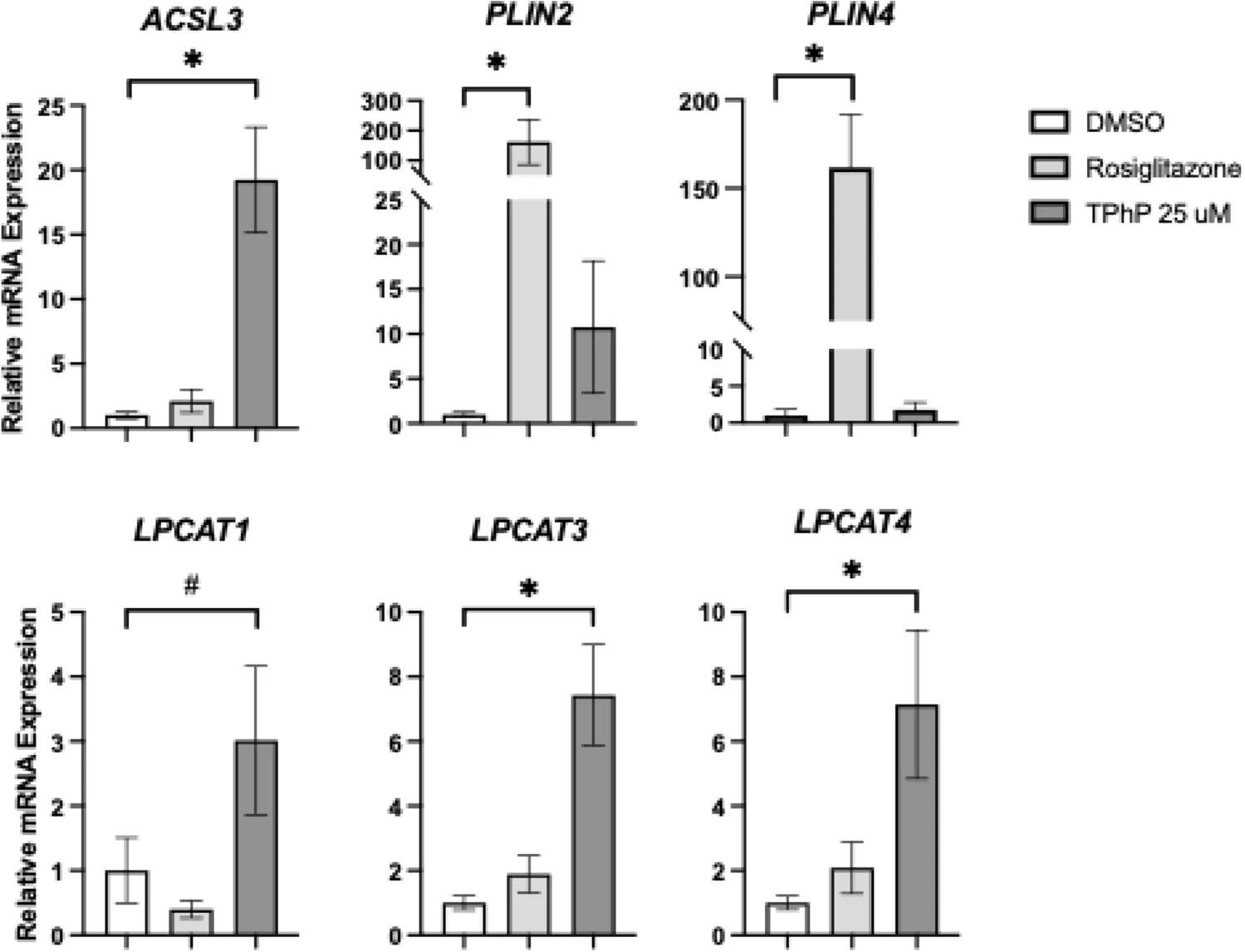
Lipid biogenesis. RNA was collected at 21 dpi and RT-qPCR was performed to measure expression changes in genes involved in the biogenesis of lipid droplets. Asterisk (*) denotes statistical significance analyzed by Student’s T test (*P* < 0.05), # denotes statistical significance analyzed by Student’s T test (*P* < 0.10) based on ODMI vs ODMI + Rosiglitazone and ODMI vs ODMI + TPhP

**Fig. 7:**
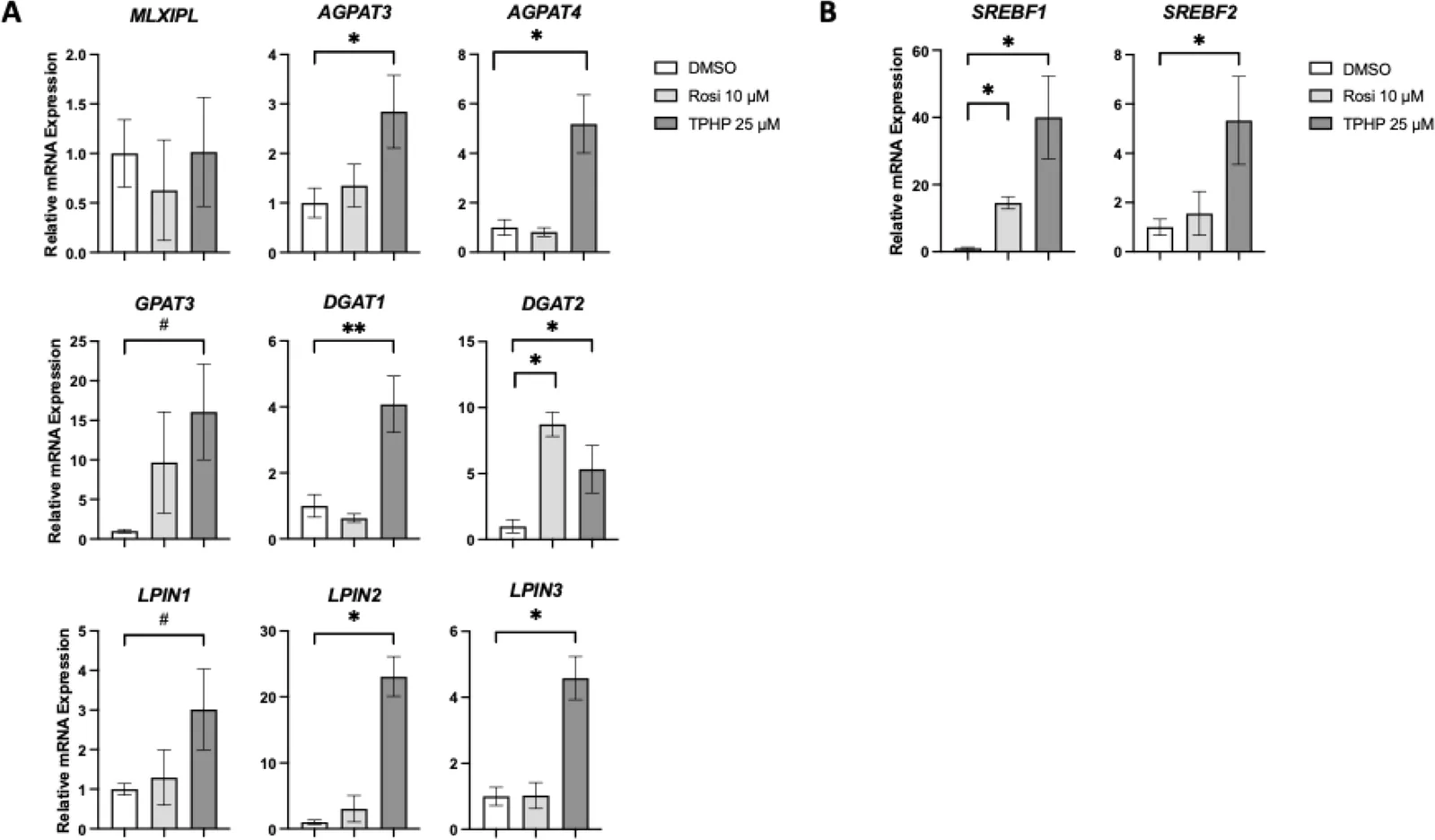
Triglyceride synthesis. RNA was collected at 21 dpi and RT-qPCR was performed to measure expression changes in genes involved in **A** triglyceride synthesis and **B** SREBF activation. Data in **A** and** B** represent mean ± SD (*n* = 3–4 technical replicates). # denote statistical significance analyzed by Student’s T test (*P* < 0.05) * (*P *< 0.01) and **(*P* <0.001) based on ODMI vs ODMI + Rosiglitazone and ODMI vs ODMI + TPhP

To dissect the metabolic pathways influenced by TPhP, we examined gene expression patterns related to lipid droplet formation, triglyceride biosynthesis, and phospholipid metabolism following 21 days of MSC differentiation. In the Rosiglitazone-treated group, expression of lipid droplet genes *PLIN2* (161.6-fold) and *PLIN4* (145.2-fold), surged consistent with its known role in promoting adipocyte maturation (Fig. 6). However, beyond this expected increase, other genes involved in lipid droplet formation remained largely unchanged. In contrast, TPhP elicited a strikingly different transcriptional profile with significant induction of of *ACSL3* (19.2-fold), *PLIN2* (10.8-fold), *LPCAT1* (3.0-fold), *LPCAT3*(7.4-fold), and *LPCAT4* (7.1-fold) (Fig. 6), indicating activation of a non-adipogenic lipid storage program. This divergent lipid signature was further supported by analysis of triglyceride biosynthesis genes (Fig. 7). While Rosiglitazone surprisingly had negligible effects on expression within this pathway with only *DGAT2* exhibiting a 18.1-fold induction, TPhP significantly upregulated key components of this pathway, including *AGPAT3* (2.8-fold)*, AGPAT4* (5.2-fold)*, GPAT3* (16.0-fold)*, DGAT1* (3.2-fold), *DGAT2* (5.3-fold), *LPIN1* (3.0-fold)*, LPIN2* (16.8-fold)*,* and *LPIN3* (7.7-fold) compared to DMSO controls. These findings suggest that TPhP promotes lipid accumulation through enhanced triglyceride synthesis, independent of traditional adipogenic cues. Further, analysis of sterol regulatory element-binding proteins (SREBPs), central regulators of lipid metabolism, revealed that Rosiglitazone selectively induced *SREBF1* (14.6-fold), while TPhP increased both *SREBF1* (40.0-fold) and *SREBF2* (5.3-fold) expression (Fig. 7), pointing to a broader and more potent activation of lipogenic transcriptional networks.

**Fig. 8:**
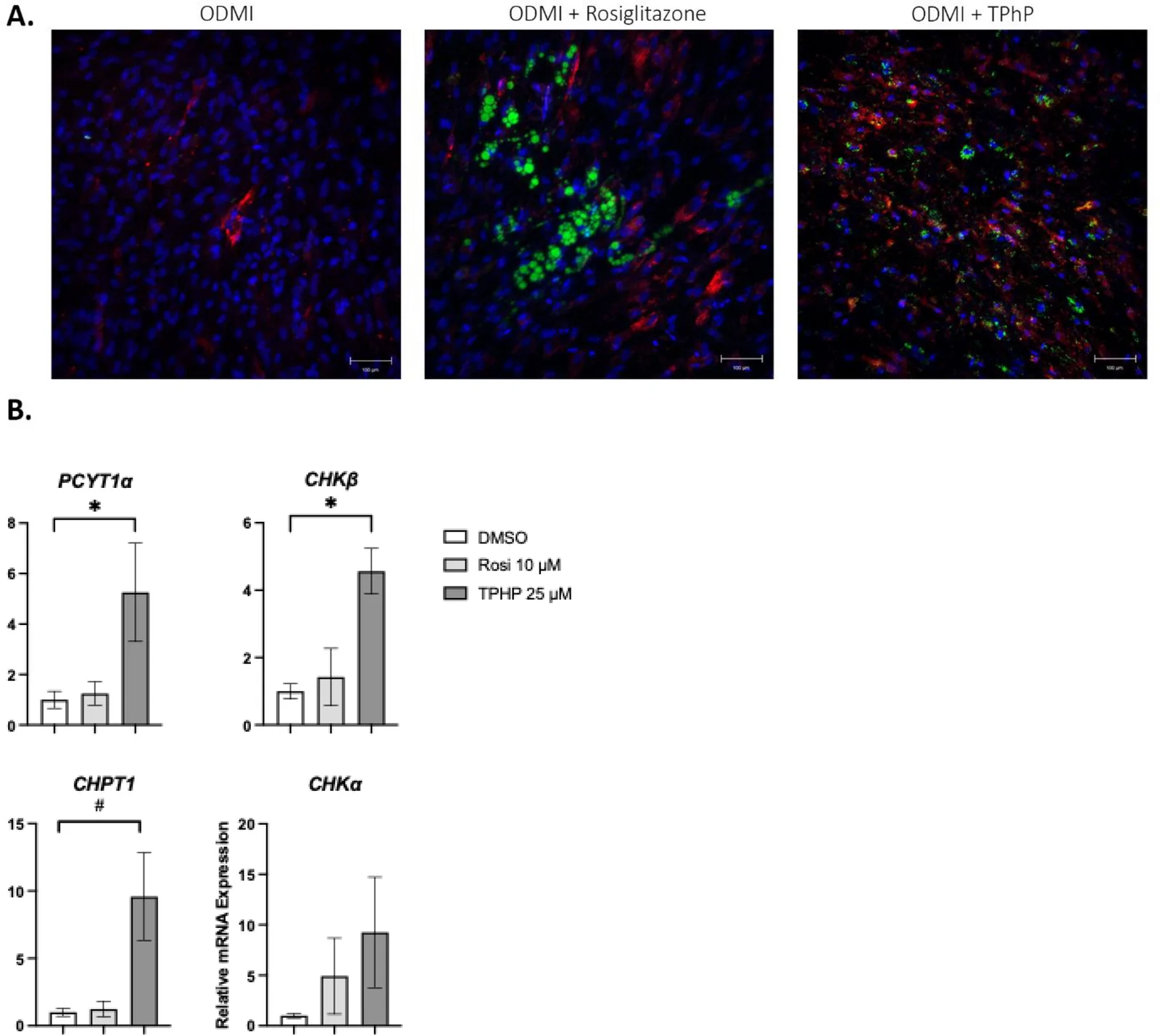
Phospholipid accumulation and the Kennedy pathway. hBMSCs were cultured in ODMI, ODMI + Rosiglitazone (10µM), and ODMI + TPhP (25µM) for 21 days. **A** Neutral lipid (Green), nuclear (Blue), and phospholipid (Red) staining was performed at 21 dpi. **B** RNA was collected at 21 dpi and RT-qPCR was performed to measure expression changes in genes involved in phospholipid synthesis, also known as the Kennedy pathway. Data represent mean ± SD (*n* = 3–4 technical replicates). * denote statistical significance analyzed by Student’s T test (*P* < 0.05), # denotes statistical significance analyzed by Student’s T test (*P* < 0.10) based on ODMI vs ODMI + Rosiglitazone and ODMI vs ODMI + TPhP

We next investigated whether TPhP influences phospholipid biosynthesis via the Kennedy pathway, a critical route for membrane lipid production that, when dysregulated, can lead to phospholipidosis (Anderson & Borlak 2006). Phospholipids are key components of the cell membrane where diglycerides (DGs) serve as the source building block and are coupled with CDP-choline to synthesize phospholipids. To visualize intracellular phospholipid accumulation, hBMSCs were stained on day 21 with HCS LipidTOX Red Phospholipidosis Detection Reagent, NucBlue for nuclei, and AdipoRed to detect neutral lipids (Fig. 8). In DMSO-treated cells, phospholipid staining was minimal, with few cells showing evidence of lipid accumulation and even fewer displaying neutral lipid content. In contrast, Rosiglitazone-treated cells exhibited abundant staining for both phospholipids and neutral lipids. Interestingly, these lipid signals appeared in distinct cell populations where neutral lipid accumulation was observed in cells with an adipocyte-like morphology, while phospholipid staining was detected in a separate set of cells, suggesting that phospholipidosis was occurring predominantly in osteoblasts rather than adipocytes. Cells treated with TPhP presented a different lipid profile. Specifically, cells treated with TPhP exhibited the diffuse, punctate neutral lipid phenotype, but, unlike the Rosiglitazone treatment group, they also exhibited diffuse and overlapping phospholipid staining throughout the population. Many cells fluoresced with both lipid markers, indicating concurrent accumulation of neutral lipids and phospholipids. This pattern suggests that TPhP induces a lipid-rich phenotype in osteoblasts or osteoblast-like cells, distinct from the adipogenic differentiation observed with Rosiglitazone. Consistent with staining for phospholipid, qPCR assessment suggests that while Rosiglitazone had little significant impact on key components of the Kennedy pathway, TPhP treatment led to marked upregulation of *CHKβ* (4.6-fold), *PCYT1α* (5.3-fold), and *CHPT1* (9.6-fold) (Fig. 8), indicating activation of phospholipid synthesis. These changes suggest a potential for TPhP to induce phospholipidosis by driving excess intracellular phospholipid accumulation.

Together, these data reveal that TPhP orchestrates a broad reprogramming of lipid metabolism in MSCs, activating multiple pathways that extend beyond adipogenic differentiation, highlighting its capacity to disrupt lipid homeostasis and contribute to pathological lipid accumulation.

### Global lipidomic profiling reveals distinct lipid signatures following TPhP and rosiglitazone exposure

To further characterize the metabolic consequences of Rosiglitazone and TPhP exposure, we conducted global, non-targeted lipidomic profiling of Rosiglitazone and TPhP treated hBMSCs. This approach enabled a comprehensive assessment of treatment-induced alterations in cellular lipid composition across multiple lipid classes. Principal component analysis (PCA) revealed clear separation between treatment and control groups, as well as distinct clustering between Rosiglitazone and TPhP-treated cells in both positive and negative ionization modes (Supplemental Fig. 4). The first two principal components (PC1 and PC2) accounted for 64.3% of the variance in positive mode and 72.9% in negative mode, highlighting robust treatment-specific shifts in the cellular lipidome. Differential abundance analysis identified numerous significantly altered lipid species, with volcano plots illustrating both upregulated and downregulated lipids in response to each treatment (log2 fold change ≥ 0.6 or ≤ –0.6; *p* ≤ 0.05) (Supplemental Fig. 4). Hierarchical clustering further demonstrated treatment-specific lipid profiles, with distinct grouping of DMSO, TPhP, and Rosiglitazone samples (Supplemental Fig. 5). To examine specific trends in lipid class composition, we focused on triacylglycerols (TGs) and phosphatidylcholines (PCs). Both treatments significantly increased TG levels relative to DMSO controls; however, Rosiglitazone induced a more uniform and robust increase across TG subclasses (Supplemental Fig. 5). In contrast, TPhP-treated cells showed a marked enrichment in PCs, aligning with earlier findings of phospholipid accumulation and activation of the CDP-choline/Kennedy pathway (Supplemental Fig. 5). Further analysis of additional phospholipid classes including phosphatidylethanolamines (PEs), phosphatidylglycerols (PGs), phosphatidylinositols (PIs), and phosphatidylserines (PSs), revealed significant increases in response to TPhP, with levels exceeding both DMSO and Rosiglitazone-treated cells (Supplemental Fig. 5). Interestingly, Rosiglitazone treatment appeared to suppress these lipid classes, emphasizing the divergent lipid regulatory mechanisms activated by each compound.

### TPhP induces hallmarks of stress-adaptive lipid remodeling

**Fig. 9:**
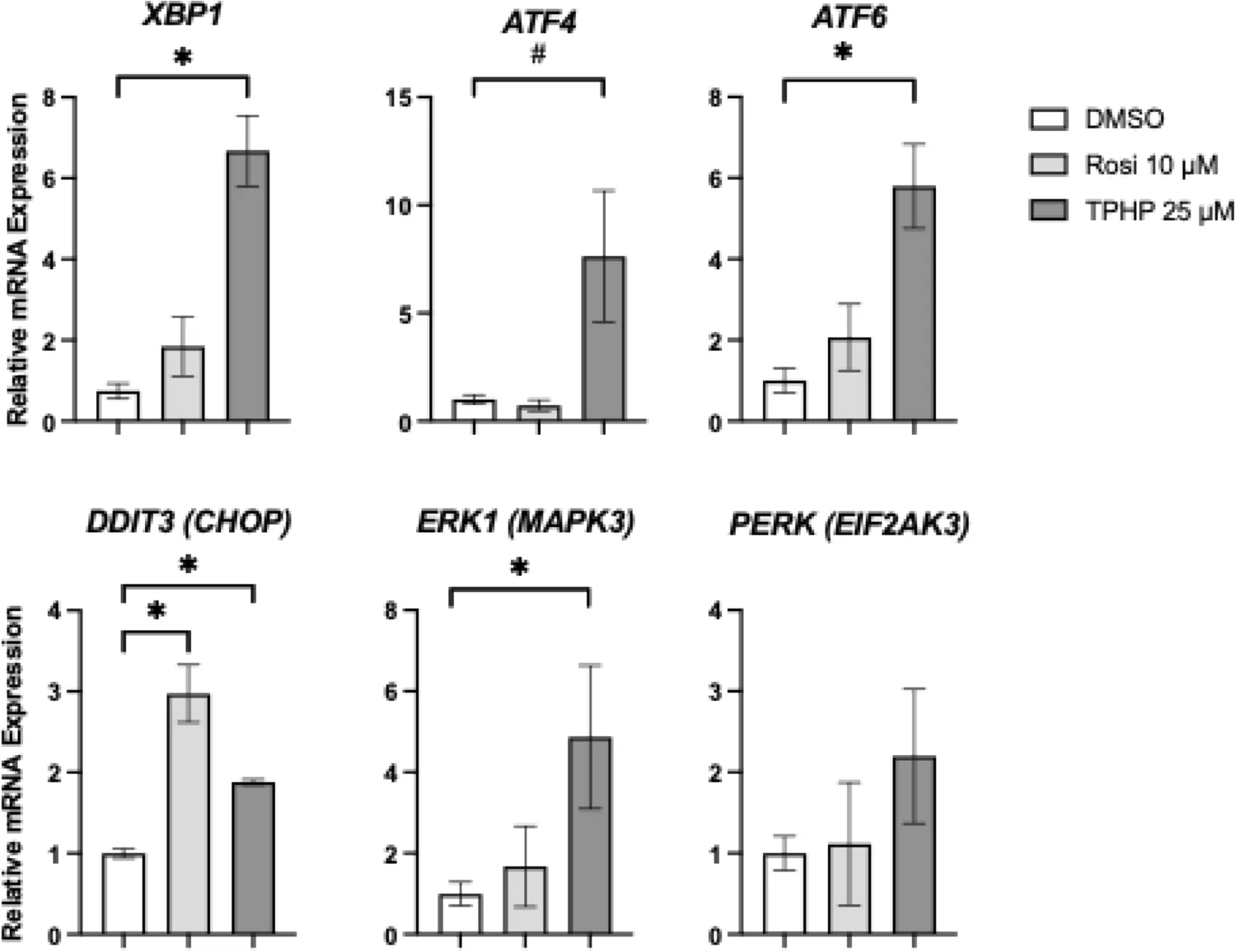
Endoplasmic Reticulum stress. hBMSCs were cultured in ODMI, ODMI + Rosiglitazone (10µM), and ODMI + TPhP (25µM) for 21 days. RNA was collected at 21 dpi and RT-qPCR was performed to measure expression changes in genes involved in the induction and regulation of endoplasmic reticulum stress. Data represent mean ± SD (*n* = 3–4 technical replicates). Asterisk (*) denotes statistical significance analyzed by Student’s T test (*P* < 0.05) and # (*P* < 0.10) based on ODMI vs ODMI + Rosiglitazone and ODMI vs ODMI + TPhP

The endoplasmic reticulum (ER) plays a central role in maintaining lipid homeostasis, particularly under stress conditions when cells must adapt to increased demands for membrane synthesis, energy regulation, and detoxification of excess or misprocessed lipids (Volmer & Ron 2015; Moncan et al. 2021). To test if TPhP exposures activate ER stress and the UPR, we performed RT-qPCR analysis targeting key regulators of the ER stress response following 21 days of exposure (Fig. 9). Rosiglitazone, a PPARγ agonist known to drive adipogenic lipid accumulation, modestly elevated the expression of *DDIT3* (CHOP) by 3.0-fold relative to DMSO controls, indicating limited activation of ER stress under these conditions. In contrast, TPhP exposure led to a robust induction of multiple UPR-associated genes. Specifically, we observed significant upregulation of *XBP1* (6.7-fold), a transcription factor activated through IRE1-mediated splicing that promotes ER membrane expansion and lipid synthesis; *ATF4* (7.6-fold), a downstream target of PERK signaling involved in stress-adaptive gene expression; *ATF6* (5.8-fold), which coordinates chaperone production and lipid metabolic regulation; *DDIT3* (CHOP) (1.9-fold), associated with prolonged ER stress and pro-apoptotic signaling; and *ERK1*(MAPK3) (4.9-fold), a kinase involved in integrating stress responses and metabolic adaptation. These findings suggest that TPhP exposure triggers a broad ER stress response and activates all three major arms of the UPR, distinguishing it from the more selective response observed with Rosiglitazone.

## Discussion

This study provides mechanistic insight into how the organophosphate ester triphenyl phosphate (TPhP) disrupts the differentiation and metabolic programming of human mesenchymal stem cells (hMSCs) via induction of endoplasmic reticulum (ER) stress. While previous studies have characterized TPhP as a PPARγ agonist capable of stimulating adipogenesis in embryonic and preadipocyte models (Pillai et al. 2014), our findings reveal a distinct, PPARγ-independent pathway in committed hMSCs undergoing osteogenic differentiation. Specifically, we show that TPhP promotes aberrant lipid accumulation likely through activation of the unfolded protein response (UPR), rather than by redirecting lineage commitment toward adipogenesis. This disruption of metabolic homeostasis is evidenced by upregulation of ER stress markers and lipid droplet-associated genes, in the absence of highly induced classical adipogenic transcriptional program.

A complex relationship exists between bone and adipose tissues, with significant crosstalk and feedback mechanisms that influence processes such as bone remodeling, adipogenesis, and energy homeostasis (Gkastaris et al. 2020). These interactions are increasingly relevant given the global health burden of obesity and osteoporosis, two conditions historically viewed as separate but now understood to be intricately linked (Palermo et al. 2016). Obesity, characterized by excessive lipid storage, and osteoporosis, marked by reduced bone mass, may share common etiological pathways including nutritional, genetic, and environmental factors (Gkastaris et al. 2020; Rinonapoli et al. 2021). Notably, metabolic disrupting chemicals (MDCs) such as TPhP are emerging as critical contributors to these disease intersections, suggesting a need to explore their effects in pluripotent systems like hMSCs, which have the potential to differentiate into both adipogenic and osteogenic lineages.

Previous studies using mouse mesenchymal stem cells (mMSCs) and 3T3-L1 cells show that TPhP is pro-adipogenic, inducing triglyceride accumulation and upregulating adipogenic markers like Fabp4 and Plin1 (Kim et al. 2020; Pillai et al. 2014; Tung et al. 2017). Mechanistically, TPhP has been shown to act as a selective PPARγ ligand, driving transcriptional activation of adipogenic pathways (Kassotis et al. 2017; Kim et al. 2020; Pillai et al. 2014). As the master regulator of adipogenesis, PPARγ promotes lipid accumulation and adiposity by reprogramming adipocyte differentiation, enhancing fat storage, and disrupting neuroendocrine control of appetite and satiety (Anghel & Wahli 2007; Lee & Ge 2014). Chemicals activating the PPARγ:RXR complex are well-recognized as adipogenic impacting lipid and adipose homeostasis (Mohajer et al. 2021).

In this study, we illustrate that TPhP exposure during osteogenic differentiation leads to a distinct lipid phenotype in hMSCs compared to other described models. Unlike Rosiglitazone, which induces robust PPARγ activation and classical adipogenic differentiation including formation of large lipid droplets and upregulation of adipogenic genes (*FABP4, LPL, PLIN1*), in this study TPhP exposure results in small, perinuclear lipid vesicles and modest or absent induction of adipocyte-specific transcriptional markers. This divergent phenotype is not attenuated by co-exposure with PPARγ antagonists (GW9662 and T0070907) compared to the phenotype observed with Rosiglitazone, suggesting putative PPARγ independence of TPhP’s effects. Immunocytochemistry supports this distinction, showing that while Rosiglitazone-treated cells are positive for the adipocyte marker *PLIN1* and exhibit a classic adipocyte morphology, TPhP-treated cells retain expression of the osteogenic marker *DLX5* and lack *PLIN1* staining indicating that lipid accumulation occurs within osteoblast-like cells.

Consistent with previous reports, our findings support that Rosiglitazone, a PPARγ agonist, promotes the browning of white adipose tissue (WAT) through the formation of “brite” (brown-in-white) adipocytes, whereas TPhP does not (Kim et al. 2020). Brite adipocytes retain select WAT-specific markers but functionally resemble brown adipose tissue (BAT), contributing to anti-obesity and antidiabetic effects; thus, induction of WAT browning is considered a therapeutic target (Giralt & Villarroya 2013; Lo & Sun 2013). In mice, full PPARγ agonists are required to activate the brown-fat gene program in subcutaneous WAT via three core regulators including PGC-1α, UCP1, and PRDM16 (Lo & Sun 2013; Ohno et al. 2012; Z. Wu et al. 1999). In our study, Rosiglitazone significantly increased PGC-1α expression at day 14 and UCP1 expression at day 21, likely through PPARγ activation, promoting mitochondrial biogenesis that underlies BAT function and metabolic benefits (Lo & Sun 2013; Z. Wu et al. 1999). In contrast, TPhP exposure resulted in a significant increase in PGC-1α at day 14 but only a negligible rise in UCP1 at day 21, suggesting it does not promote browning in hMSCs under osteogenic conditions. While these results align with Kim et al. (2020) in showing that TPhP fails to induce brite adipogenesis, they differ from prior conclusions that TPhP favors white adipogenesis, at least within an osteogenic context. Notably, TPhP exposure produced a mixed pattern of up- and downregulation among BAT regulators and yielded small, diffuse multilocular lipid droplets, morphologically distinct from the unilocular droplets characteristic of WAT and the large multilocular droplets typical of BAT or brite adipocytes.

Global lipidomics analysis further supports this divergence, revealing that TPhP and Rosiglitazone induce distinct lipid profiles. Rosiglitazone predominantly elevated triacylglycerol (TG) levels, consistent with its adipogenic role, whereas TPhP significantly increased neutral lipids and phospholipids, including phosphatidylcholines, phosphatidylethanolamines, phosphatidylglycerols, phosphatidylinositol, and phosphatidylserine. This phospholipid enrichment coincided with activation of the Kennedy pathway and the development of phospholipidosis, observed through fluorescent staining. These findings align with prior reports demonstrating that TPhP and other aryl-OPFRs disrupt lipid metabolism in HepG2 cells and in vivo mouse models (An et al. 2023; Hao et al. 2019; D. Wang et al. 2019). The upregulation of Kennedy pathway genes, coupled with intracellular phospholipid accumulation, suggests that TPhP promotes lipid biosynthesis pathways typically associated with ER function and membrane synthesis, rather than those supporting adipocyte differentiation and maturation in this hMSC’s model (Qi et al. 2017).

Endoplasmic reticulum (ER) stress is a critical regulator of cellular lipid metabolism, particularly under conditions of metabolic or toxicological stress. When the folding capacity of the ER is overwhelmed, misfolded or unfolded proteins accumulate, triggering the unfolded protein response (UPR), which aims to restore ER function through a coordinated transcriptional and translational program (Hetz & Papa 2018; Ron & Walter 2007). Three primary sensors, IRE1α, PERK, and ATF6, mediate this response and have been shown to play key roles in lipid homeostasis (Malhotra & Kaufman 2007). Notably, activation of the IRE1α-XBP1s axis promotes expression of genes involved in phospholipid and triglyceride biosynthesis, including stearoyl-CoA desaturase (SCD), diacylglycerol acyltransferase (DGAT), and specific enzymes within the Kennedy pathway (Moncan et al. 2021; Sriburi et al. 2007; Volmer & Ron 2015). Additionally, UPR signaling can activate sterol regulatory element-binding proteins (SREBPs), which induce subsequent lipogenic network (Bobrovnikova-Marjon et al. 2008; Hamanaka et al. 2009). In this study, exposure to TPhP resulted in significant upregulation of SREBP1/2 and key regulators of ER stress including *XBP1*, *ATF4*, ATF6, *ERK1* and *CHOP*. These pathways collectively drive lipid droplet formation, which serves as a cytoprotective mechanism to sequester excess or toxic lipids, thus preserving membrane integrity and limiting lipotoxicity (Olzmann & Carvalho 2019; Walther et al. 2017). However, chronic ER stress can lead to excessive lipid accumulation, contributing to metabolic pathologies such as non-alcoholic steatohepatitis (NASH), insulin resistance, and obesity (Hotamisligil 2010; Lebeaupin et al. 2018; Ma et al. 2024; Na et al. 2023). In progenitor cells such as mesenchymal stem cells, ER stress and in some instances lipid biogenesis can also interfere with differentiation programs, shifting cellular fate and function in ways that compromise tissue repair and homeostasis (Han & Kaufman 2016; Saito et al. 2011; Tavasolian et al. 2020). The decoupling of lipid accumulation from adipogenic differentiation is increasingly recognized in models of metabolic stress and toxicant exposure (Lebeaupin et al. 2018; Li et al. 2023), reinforcing our interpretation of TPhP’s non-classical mechanism. The lipid accumulation in TPhP-treated hMSCs may thus represent an adaptive, stress-induced storage response to limit cytotoxic damage from free fatty acids or misfolded proteins and underscore the dual role of ER stress in both maintaining and disrupting lipid equilibrium, with significant implications for disease progression and environmental toxicant response.

Importantly, ER stress has previously been implicated in impaired osteogenesis and is a known contributor to skeletal aging and osteoporosis (Iyer et al. 2023; T. Wu et al. 2023). The unfolded protein response is tightly linked to osteoblast function, where persistent activation can compromise bone-forming capacity (Horiuchi et al. 2016; Zhong et al. 2023). In particular, prolonged or excessive activation of UPR signaling elements such as XBP1, ATF4, and CHOP has been associated with reduced osteoblast survival and impaired differentiation (Murakami et al. 2009; Turishcheva et al. 2022). TPhP-induced activation of these UPR components may therefore hinder osteoblast maturation and mineralization, as supported by our observation of reduced extracellular matrix (ECM) deposition despite preserved expression of canonical osteogenic markers such as RUNX2 and OSX. This phenotype, characterized by disrupted matrix mineralization in the absence of complete suppression of osteogenic transcriptional programs, suggests that metabolic interference, rather than lineage commitment failure, is a key mechanism. Such metabolic disruption may include altered redox balance, ATP depletion, or as observed, dysregulated lipid handling, which are known to accompany ER stress and can critically impair the energy intensive process of matrix synthesis and mineralization (Ghemrawi et al. 2018; Malhotra & Kaufman 2007; Riddle & Clemens 2017). Collectively, these findings indicate that TPhP exposure shifts the metabolic balance of human mesenchymal stem cells (hMSCs) under osteogenic conditions, which may lead to dysfunctional bone remodeling and potentially contribute to long-term skeletal deficits, particularly in the context of chronic environmental exposures.

Our findings advance the recognition of organophosphate esters (OPEs) such as triphenyl phosphate (TPhP) as metabolic disruptors that act through diverse mechanisms beyond classical endocrine disruption. TPhP notably is demonstrated to induce lipid accumulation, disrupt mitochondrial function, stimulate PPARγ and SREBP2 driven lipogenesis, and trigger ER stress in select cell types including human mesenchymal stem cells (hMSCs) undergoing osteogenic differentiation (Pillai et al. 2014; An et al. 2023; Chen et al. 2024; Hao et al. 2019). Aberrant lipid storage under bone-forming conditions suggests potential interference with osteoblastogenesis, predisposing to ectopic fat deposition in the bone marrow niche and amplifying risk for age related skeletal loss (Ambrosi et al. 2017; Justesen et al. 2001; Pierce et al. 2019). The ability of TPhP to impair autophagy/lipophagy further exacerbates intracellular lipid retention (Li et al. 2023; Yue et al. 2023), while alternative signaling routes, such as GPER-mediated PI3K/Akt and PPAR cascades, have emerged as non-classical pathways contributing to OPE-driven metabolic changes (Guan et al. 2022). Collectively, these observations underscore how environmental xenobiotics can rewire multipotent stem cell fate via energy sensing and microenvironmental pathways, uncoupling lineage specification from lipid homeostasis and revealing broader metabolic and skeletal health implications.

This work also highlights the need to expand toxicological screening frameworks beyond PPARγ activation and adipocyte marker expression. As shown here, TPhP elicits profound effects on lipid metabolism, ER homeostasis, and stem cell function through non-canonical pathways. Incorporating assessments of organelle stress, metabolic reprogramming, and lipid droplet dynamics in stem cell models may offer more sensitive and predictive tools for identifying metabolic disruptors. Given the ubiquity of TPhP, and many structural analogues of TPhP (differing in methyl, ethyl or propyl groups on each ring), in consumer products and its functional overlap with legacy flame retardants, our findings underscore the need for rigorous safety evaluations of current-use organophosphates and their long-term impacts on human health. Future research should also investigate the dose-dependency of these effects and long-term consequences of chronic, low-dose exposure in both in vitro and in vivo systems. While concentrations utilized in this study are generally higher than those found in human blood or urine, in vitro and developmental model systems often require micromolar concentrations to account for rapid metabolism, limited bioavailability, and the absence of long-term accumulation processes present in vivo. Thus, concentrations utilized in this study ensure robust induced biological effects while remaining below thresholds associated with nonspecific toxicity, allowing for clear interpretation of pathway-level and mechanistic outcomes relevant to hazard identification and risk assessment.

## Conclusions

In summary, TPhP disrupts osteogenic differentiation in human MSCs by inducing ER stress and triggering aberrant lipid accumulation via non-classical, PPARγ-independent mechanisms. These effects result in the formation of lipid laden osteoblast-like cells, reduced extracellular matrix formation, and altered lipid metabolism without promoting terminal adipogenesis. Our findings highlight a novel mechanism by which environmental MDCs like TPhP can impair skeletal homeostasis and underscore the need to evaluate toxicants using broader mechanistic frameworks. This work expands our understanding of how cellular stress responses intersect with differentiation programs in stem cells and suggests that dysregulated lipid handling, rather than classical adipogenic conversion, may underlie some toxicant-induced metabolic disorders.

## Supplementary Information

Below is the link to the electronic supplementary material.

Supplementary file1 (DOCX 6617 kb)
