# Supplementary material for: Triphenyl phosphate promotes lipid accumulation in human mesenchymal stem cells through metabolic stress pathways
Source: Arch Toxicol. 2026 Jun 2;100(10):4645–65. doi: 10.1007/s00204-026-04445-5 (PMC13593771; doi:10.1007/s00204-026-04445-5)
Supplement: Supplementary file 1 [file 204_2026_4445_MOESM1_ESM.docx]

**SUPPLEMENTAL DATA**

**TABLES**

| **Supplemental Table 1. Primer sequences for adipogenic and BAT marker related qPCR.** | |
| --- | --- |
| **Gene** | **Primer Sequence (5’ – 3’)** |
| ***PPARγ*** | GATACACTGTCTGCAAACATATCACAA (forward) |
|  | CCACGGAGCTGATCCCAA (reverse) |
| ***FABP4*** | CCTTTAAAAATACTGAGATTTCCTTCA (forward) |
|  | GGACACCCCCATCTAAGGTT (reverse) |
| ***C/EBPα*** | AAGAAGTCGGTGGACAAGAACAG (forward) |
|  | TGCGCACCGCGATGT (reverse) |
| ***PLIN*** | TGTGCAATGCCTATGAGAAGG (forward) |
|  | AGGGCGGGGATCTTTTCCT (reverse) |
| ***LPL*** | TCATTCCCGGAGTAGCAGAGT (forward) |
|  | GGCCACAAGTTTTGGCACC (reverse) |
| ***LEP*** | ATGACACCAAAACCCTCATCAA (forward) |
|  | GAAGTCCAAACCGGTGACTTT (reverse) |
| ***ADIPOQ*** | CCCTCTCTTACAAGCCCATCA (forward) |
|  | GAGCCAGTCTGGTAGTACATCA (reverse) |
| ***TRPV1*** | GGCTGTCTTCATCATCCTGCTGCT (forward) |
|  | GTTCTTGCTCTCCTGTGCGATCTTGT (reverse) |
| ***PGC1α*** | GCCAAACCAACAACTTTATCTCTTC (forward) |
|  | CACACTTAAGGTGCGTTCAATAGTC (reverse) |
| ***UCP1*** | CTGGAATAGCGGCGTGCTT (forward) |
|  | AATAACACTGGACGTCGGGC (reverse) |
| ***PRDM-16*** | AAAGAGGCGCAGTTCCCAAT (forward) |
|  | GGGCACCGAGGCTTAGAAAA (reverse) |

**Supplemental Table 1.** Primer sequences for adipogenic and BAT marker related qPCR.

| **Supplemental Table 2. Primer sequences for osteogenic transcription factor related qPCR.** | |
| --- | --- |
| **Gene** | **Primer Sequence (5’ – 3’)** |
| ***BMP4*** | ACGGTGGGAAACTTTTGATGTG (forward) |
|  | CGAGTCTGATGGAGGTGAGTC (reverse) |
| ***TWIST*** | CCAGGTACATCGACTTCCTCTA (forward) |
|  | CCATCCTCCAGACCGAGAA (reverse) |
| ***RUNX2*** | TGGTTACTGTCATGGCGGGTA (forward) |
|  | TCTCAGATCGTTGAACCTTGCTA (reverse) |
| ***OSX/SP7*** | GCCAGAAGCTGTGAAACCTC (forward) |
|  | TCTCCATAACCATGGCAACA (reverse) |

**Supplemental Table 2.** Primer sequences for osteogenic transcription factor related qPCR.

| **Supplemental Table 3. Primer sequences for lipid biogenesis, triglyceride synthesis, and Kennedy pathway related qPCR.** | |
| --- | --- |
| **Gene** | **Primer Sequence (5’ – 3’)** |
| ***ACSL3*** | CAAGGGCATCATTGTGCAT (forward) |
|  | GGGCAATGGTTTGCTATGAG (reverse) |
| ***PLIN2*** | CATTGTGTTGCCTCTGTTAC (forward) |
|  | CCTTTTCAGATCACACCAGA (reverse) |
| ***PLIN4*** | CCTGGACCACTCCCTTAG (forward) |
|  | CAACCTTCGGAAAAGCAGAT |
| ***LPCAT1*** | CCTCATGACACTGACGCTCTTC (forward) |
|  | CAGGAAGTCCACAACCTTCCTC (reverse) |
| ***LPCAT3*** | ATACTATTACACTGCCACCGGCAA (forward) |
|  | AGTAGGAGAAACCAGCAACTTCCA (reverse) |
| ***LPCAT4*** | CTGCAGCATTGTGGATGTGG (forward) |
|  | TGTGCCATGACCCTCTGAAC (reverse) |
| ***MLXIPL*** | TGTGCTGGTCAGTGTCTCAC (forward) |
|  | GCTGCTTCCAGCTCAGAGTA (reverse) |
| ***AGPAT3*** | CTCCAAGGTCCTCGCTAAGAAG (forward) |
|  | CCGCTTGCAGAACACAATCTC (reverse) |
| ***AGPAT4*** | CACGGAATGCACCATCTTCA (forward) |
|  | GAACCACGATGGCATTTTCCT (reverse) |
| ***GPAT3*** | CAAGGAGGCCTGACTGAACTTCCC (forward) |
|  | CCGTCCTCTTAGCTGAGAGATCCATTG (reverse) |
| ***DGAT1*** | CAACAAGGACGGAGACGCCGG (forward) |
|  | GATGCCACGGTAGTTGCTGAAGCC (reverse) |
| ***DGAT2*** | CTGCACTGATTGCTGGCTCATCG (forward) |
|  | GAAAGTAGCGCCACACAGCCCAG (reverse) |
| ***LPIN1*** | TCTGGGGTGTGTTATCAAGCA (forward) |
|  | GGGAACCCCCATGTGCTAAA (reverse) |
| ***LPIN2*** | CAGACCCTGTCCTTTTCCCC (forward) |
|  | GTCCCATCACAGGGCTCAAA (reverse) |
| ***LPIN3*** | GATGGCTCTATGCCACCCTG (forward) |
|  | CGGGCTTGTATGTGTAGGGG (reverse) |
| ***SREBF1*** | TTCTGTGAAAGGCCGACTCC (forward) |
|  | TGCTTCACACTGAAGATTGTGT (reverse) |
| ***SREBF2*** | CTCCCACATCTGGCATTGGT (forward) |
|  | AACTTGTCCAGCCTCTCACG (reverse) |
| ***CHKα*** | CTGGGGACTGTGGTCCATTG (forward) |
|  | TGGGGAGACAGAGAACCCTC (reverse) |
| ***CHKβ*** | TTGGTAAGTGACCCTGGGGA (forward) |
|  | GTGAGAAGACGTGGGACCAG (reverse) |
| ***PCYT1α*** | ATTGAGCAGGAAGGGGCATC (forward) |
|  | GATAAAAGCTGGGCAAGGCG (reverse) |
| ***CHPT1*** | AGGACCACTTTTCATCTCAGGT (forward) |
|  | AGGAAACCCATTGTGGGCAA (reverse) |

**Supplemental Table 3.** Primer sequences for lipid biogenesis, triglyceride synthesis, and Kennedy pathway related qPCR.

| **Supplemental Table 4. Primer sequences for ER stress related qPCR.** | |
| --- | --- |
| **Gene** | **Primer Sequence (5’ – 3’)** |
| ***XBP1*** | TGTAAGCAACGGGAACACCT (forward) |
|  | AGCGTTAGGCTCACTTCAGTT (reverse) |
| ***ATF4*** | GTCCAAGGCTGAAGATCGGT (forward) |
|  | TGTTGGCATGAAGCCCTCTT (reverse) |
| ***ATF6*** | TGCTCAGGTTGTACCCATGC (forward) |
|  | AGCCACAAAGCCTTTTCAGG (reverse) |
| ***DDIT3***  ***(CHOP)*** | GCGTGCCACTTTCTGATTGG (forward) |
|  | AGCGCCTGGCTGTAATCTTT (reverse) |
| ***ERK1***  ***(MAPK3)*** | CAGACATCTCTGCACCCTGG (forward) |
|  | GGGGTAAGCTTGCTGCTGTA (reverse) |
| ***PERK***  ***(EIF2AK3)*** | TCCTAACCCATTTCAGACAGGT (forward) |
|  | AACTAGCATCACCACAGCCC (reverse) |

**Supplemental Table 4.** Primer sequences for mitochondrial and ER stress related qPCR.

| **Supplemental Table 5. Summary qPCR Fold Change and SEM** | | | | | | | |
| --- | --- | --- | --- | --- | --- | --- | --- |
| **Gene name** | **Figure** | **DMSO (Normalized)** | **SEM** | **Rosiglitazone** | **SEM** | **TPhP** | **SEM** |
| **IBSP** | **2** | 1.000 | 0.367 | 1.584 | 0.111 | 0.091 | 0.014 |
| **SSP1** |  | 1.000 | 0.218 | 1.120 | 0.074 | 0.420 | 0.041 |
| **BGLAP** |  | 1.000 | 0.135 | 0.676 | 0.068 | 1.297 | 0.133 |
|  |  |  |  |  |  |  |  |
| **PPARy-14** | **3** | 1.000 | 0.212 | 3.582 | 0.340 | 0.558 | 0.071 |
| **FABP-14** |  | 1.000 | 0.289 | 845.176 | 20.233 | 0.161 | 0.038 |
| **C/EBPa-14** |  | 1.000 | 0.160 | 1.231 | 0.318 | 0.712 | 0.010 |
| **PLIN1-14** |  | 1.000 | 0.106 | 18.612 | 3.148 | 1.489 | 0.262 |
| **LPL-14** |  | 1.000 | 0.058 | 162.553 | 13.041 | 1.630 | 0.622 |
| **LEP-14** |  | 1.000 | 0.259 | 0.765 | 0.152 | 0.286 | 0.107 |
| **PGC1a-14** |  | 1.000 | 0.635 | 28.690 | 2.895 | 27.436 | 1.590 |
| **UCP-14** |  | 1.000 | 0.229 | 1.574 | 0.225 | 1.126 | 0.148 |
|  |  |  |  |  |  |  |  |
| **PPARy-21** |  | 1.000 | 0.407 | 18.164 | 3.743 | 3.336 | 1.004 |
| **FABP-21** |  | 1.000 | 0.455 | 3674.937 | 146.122 | 10.596 | 1.475 |
| **C/EBPa-21** |  | 1.000 | 0.203 | 2.929 | 0.732 | 1.764 | 0.126 |
| **PLIN1-121** |  | 1.000 | 0.085 | 101.196 | 12.134 | 2.402 | 0.325 |
| **LPL-21** |  | 1.000 | 0.494 | 832.167 | 157.200 | 2.796 | 1.247 |
| **LEP-21** |  | 1.000 | 0.353 | 1.900 | 0.301 | 1.992 | 0.230 |
| **PGC1a-21** |  | 1.000 | 0.226 | 1.662 | 0.197 | 2.431 | 1.125 |
| **UCP-21** |  | 1.000 | 0.382 | 48.860 | 8.390 | 3.246 | 1.044 |
|  |  |  |  |  |  |  |  |
| **TWIST** | **5** | 1.000 | 0.172 | 1.486 | 0.466 | 9.052 | 0.732 |
| **OSX** |  | 1.000 | 0.237 | 1.911 | 0.695 | 14.049 | 2.096 |
| **RUNX2** |  | 1.000 | 0.141 | 0.298 | 0.060 | 4.390 | 2.037 |
|  |  |  |  |  |  |  |  |
| **ACSL3** | **6** | 1.000 | 0.256 | 2.419 | 2.500 | 19.236 | 4.090 |
| **PLIN2** |  | 1.000 | 0.263 | 161.550 | 11.320 | 10.771 | 1.850 |
| **PLIN4** |  | 1.149 | 0.545 | 145.280 | 8.749 | 1.397 | 0.495 |
| **LPCAT1** |  | 1.000 | 0.504 | 0.400 | 0.137 | 3.014 | 1.149 |
| **LPCAT3** |  | 1.000 | 0.218 | 1.889 | 0.575 | 7.432 | 1.563 |
| **LPCAT4** |  | 1.000 | 0.214 | 2.090 | 0.800 | 7.145 | 2.303 |
|  |  |  |  |  |  |  |  |
| **MLXIPL** | **7** | 1.000 | 0.341 | 0.628 | 0.507 | 1.017 | 0.552 |
| **AGPAT3** |  | 1.000 | 0.297 | 1.350 | 0.432 | 2.845 | 0.737 |
| **AGPAT4** |  | 1.000 | 0.318 | 0.817 | 0.172 | 5.182 | 1.175 |
| **GPAT3** |  | 1.000 | 0.205 | 9.672 | 6.410 | 16.066 | 6.049 |
| **DGAT1** |  | 1.000 | 0.343 | 0.630 | 0.128 | 3.223 | 0.988 |
| **DGAT2** |  | 1.000 | 0.504 | 18.055 | 9.337 | 5.333 | 1.801 |
| **LIPN1** |  | 1.000 | 0.149 | 1.293 | 0.694 | 3.013 | 1.025 |
| **LIPN2** |  | 1.000 | 0.349 | 3.039 | 1.996 | 16.752 | 6.578 |
| **LIPN3** |  | 1.000 | 0.280 | 1.025 | 0.394 | 7.677 | 3.119 |
| **SREBF1** |  | 1.000 | 0.274 | 14.551 | 1.809 | 40.004 | 12.366 |
| **SREBF2** |  | 1.000 | 0.329 | 1.560 | 0.891 | 5.333 | 1.791 |
|  |  |  |  |  |  |  |  |
| **PCYT1a** | **8** | 1.000 | 0.335 | 1.253 | 0.467 | 5.259 | 1.951 |
| **CHKb** |  | 1.000 | 0.227 | 1.424 | 0.849 | 4.565 | 0.676 |
| **CHPT1** |  | 1.000 | 0.290 | 1.243 | 0.572 | 9.586 | 3.258 |
| **CHKa** |  | 1.000 | 0.218 | 4.925 | 3.768 | 9.238 | 5.474 |
|  |  |  |  |  |  |  |  |
| **XBP1** | **9** | **1** | 0.19 | 1.844 | 0.733 | 6.677 | 0.875 |
| **AFT4** |  | 1 | 0.214 | 0.720 | 0.257 | 9.182 | 0.700 |
| **AFT6** |  | 1 | 0.306 | 2.070 | 0.820 | 5.795 | 1.039 |
| **DDIT3** |  | 1 | 0.059 | 2.972 | 0.355 | 1.881 | 0.034 |
| **ERK1** |  | 1 | 0.289 | 1.674 | 0.995 | 4.867 | 1.770 |
| **PERK** |  | 1 | 0.213 | 1.116 | 0.759 | 2.197 | 0.827 |
|  |  |  |  |  |  |  |  |
| **Gene name** | **Figure** | **Fold Change** | **SEM** |  |  |  | |
| **PPARy-D** | 4 | 1 | 0.107 |  |  |  | |
| **PPARy-R** |  | 53.306 | 3.94 |  |  |  | |
| **PPARy-RG** |  | 0.665 | 0.077 |  |  |  | |
| **PPARy-RT** |  | 1.295 | 0.028 |  |  |  | |
| **PPARy-T** |  | 0.646 | 0.059 |  |  |  | |
| **PPARy-TG** |  | 0.942 | 0.064 |  |  |  | |
| **PPARy-TT** |  | 2.653 | 0.286 |  |  |  | |
|  |  |  |  |  |  |  | |
| **FABP4-D** |  | 1.000 | 0.192 |  |  |  | |
| **FABP4-R** |  | 226.149 | 19.630 |  |  |  | |
| **FABP4-RG** |  | 0.831 | 0.065 |  |  |  | |
| **FABP4-RT** |  | 0.147 | 0.077 |  |  |  | |
| **FABP4-T** |  | 1.691 | 0.076 |  |  |  | |
| **FABP4-TG** |  | 0.478 | 0.093 |  |  |  | |
| **FABP4-TT** |  | 0.032 | 0.010 |  |  |  | |
|  |  |  |  |  |  |  | |
| **PLIN1-D** |  | 1.000 | 0.092 |  |  |  | |
| **PLIN1-R** |  | 11.700 | 0.574 |  |  |  | |
| **PLIN1-RG** |  | 0.276 | 0.048 |  |  |  | |
| **PLIN1-RT** |  | 0.600 | 0.037 |  |  |  | |
| **PLIN1-T** |  | 0.849 | 0.042 |  |  |  | |
| **PLIN1-TG** |  | 0.354 | 0.038 |  |  |  | |
| **PLIN1-TT** |  | 0.768 | 0.079 |  |  |  | |

D-DMSO, R-Rosiglitazone, T-TPhP, RG-Rosiglitazon+GW9662, RT-Rosiglitazone+T0070907, TG TPhP-GW9622, TT-TPhP+ T0070907.

**Supplemental Figure 1. Quantitative Resazurin cytotoxicity Assay**. Mesenchymal stem cell (hMSC) viability was assessed using a resazurin-based metabolic assay over a 21-day culture period. MSCs were seeded in 12 well tissue culture plates and maintained under standard growth conditions, with media (ODMI, +/- treatments) changes performed every 2–3 days. At 21days, cells were washed. In PBS and culture medium was replaced with fresh medium containing resazurin solution (final concentration 10% v/v of a 0.15 mg/mL stock). Cells were incubated for 2–4 hours at 37°C, protected from light. Following incubation, aliquots of the conditioned medium were transferred to a 96-well plate, and fluorescence was measured using a microplate reader (excitation ~560 nm, emission ~590 nm). Background fluorescence from cell-free controls was subtracted, and values were normalized to ODMI media. Data were interpreted as an indicator of metabolic activity and cell viability over time. Cells treated 0.1% DMSO, 10 µM Rosiglitazone, or 25 µM TPhP retained between 98-90% cell viability. 0.1% Triton X 100 was used as a positive control.

**Supplemental Figure 2.** **Top 20** **Hallmark Pathways of Normalized Enrichment Scores from Gene Set Enrichment Analysis**. Rosiglitazone vs DMSO at 21 days post osteoinduction of hMSC’s in the presence of 10uM Rosiglitazone. Gene set enrichment was conducted using (GSEA (version 4.0.3)) (Subramanian et al., 2005) as described in materials and methods.

**Supplemental Figure 3.**  **Top 20** **Hallmark Pathways of Normalized Enrichment Scores from Gene Set Enrichment Analysis**. TPhP vs DMSO at 21 days post osteoinduction of hMSC’s in the presence of 10uM Rosiglitazone. Gene set enrichment was conducted using (GSEA (version 4.0.3)) (Subramanian et al., 2005) as described in materials and methods.

**Positive mode**

**Negative mode**

**A**

**B**

**C**

**D**

**E**

**F**

**TPhP**

**TPhP**

**Rosiglitazone**

**Rosiglitazone**

**Supplemental Figure 4. Lipidomics Assessments of TPhP and Rosiglitazone exposures in human mesenchymal stem cells under osteogenic conditions.** (A, B) Principal component analysis in positive and negative ionization modes. (C-F) Differential lipid abundance analysis of compounds detected in the positive mode (C, E) and negative mode (D, F) lipidomics datasets. Compounds highlighted in volcano plots are significant at a P-value ≤ 0.05 with log2 fold change ≥ 0.6 (red) or ≤ -0.6 (green).

**Supplemental Figure 5. Observed changes in lipid profiles following treatment with ROSI or TPhP.** A. Hierarchical cluster analysis illustrates grouping of treatments by measured Triacylglycerols (TG) and Phosphatidylcholines (PC), CD-DMSO, CR-Rosiglitazone, CT-TPhP. **B**. Triglycerols profile for DMSO, Rosiglitizone and TPhP. **C.** Phosphatidylcholines profile for DMSO, Rosiglitizone and TPhP. D Additional phospholipid classes elevated by TPHP: Phosphatidylethanolamines (PE), Phosphatidylglycerols (PG), Phosphatidylinositols (PI), Phosphatidylserines (PS)**.** Data represent the mean ± standard deviation (n=4). Inset includes peak area distributions for upregulated phospholipids from each class following Rosiglitazone and TPHP exposure.
